# The use of palliative care by people of Islamic faith and their preferences and decisions at the end of life: A scoping review

**DOI:** 10.1017/S1478951525000148

**Published:** 2025-03-31

**Authors:** Ibrahim AL Shhadat, Lisa-Maria Wobst, Gabriele Meyer, Rustem Makhmutov, Steffen Fleischer

**Affiliations:** Institute of Health and Nursing Science, Medical Faculty of Martin Luther University Halle-Wittenberg, University Medicine Halle, Halle (Saale), Germany

**Keywords:** End-of-life decisions, euthanasia attitudes, palliative care, barriers and facilitators, Islam

## Abstract

**Objectives:**

The use of palliative care (PC) services from people of Islamic faith is seen limited. There are a fundamental lack of PC services appropriate to the target group and a lack of knowledge and acceptance. The transition from curative to PC is often perceived as problematic. Factors influencing PC use and end-of-life (EOL) decisions and preferences among people of Islamic faith are largely unclear.

**Methods:**

A scoping review was carried out using the methodology of the Joanna Briggs Institute. Studies of any design, published in English, German, or Arabic, and published by the end of August 2022, were eligible for inclusion. The systematic literature search was conducted in MEDLINE via PubMed, CINAHL, Cochrane Library, and Web of Science. Study statements were analyzed with a clear distinction between PC as EOL care and other EOL decisions, such as euthanasia, withdrawal, or withholding of one or more life-sustaining treatments or medications.

**Results:**

Sixty studies published between 1998 and 2022 were included. Only a few studies made statements about EOL care. The majority of studies focused on forms of euthanasia and indicated negative attitudes toward euthanasia, assisted suicide, and some other EOL decisions. Reasons for rejection include theological arguments, ethical and moral considerations, and others. Reasons for acceptance were principles of good death and dying, medical justifications, and others. The following barriers to the use of PC were identified laws and policies, lack of necessary resources, cultural norms and values, structure of the health-care system, communication and interaction between patients, relatives, and health-care staff, and others.

**Significance of results:**

This review identifies the preferences for and difficulties in making EOL decisions and identifies barriers to specific PC for the Muslim population. Findings suggest how these barriers might be overcome.

## Introduction

The World Health Organization (WHO) defines palliative care (PC) as “an approach that improves the quality of life of patients (adults and children) and their families who are facing problems associated with life-threatening illness. It prevents and relieves suffering through the early identification, correct assessment and treatment of pain and other problems, whether physical, psychosocial or spiritual” (WHO 2020a). Only 40% of countries stated that at least half of patients in need of PC received it, according to a 2019 WHO noncommunicable disease survey of 194 member states (WHO 2020b). This highlights significant gaps in access to essential PC globally. Nearly 60 million people need PC each year, with an estimated 25.7 million people needing PC in their last year of life. Most people requiring PC also live in low- and middle-income countries, many of these countries are also home to the majority of people of Muslim faith (Clark et al. 2020; WHO 2020a; WorldAtlas 2019). However, there is a significant deficit of PC services in countries with Muslim majority (Al-Awamer and Downar 2014; Weng et al. 2021). Islam holds the sanctity of human life in high esteem and considers its preservation necessary. Nevertheless, the obligation to apply life-prolonging measures may not be considered imperative when these are futile, particularly in cases of untreatable illness accompanied by significant distress and suffering. While euthanasia is strictly forbidden in Islamic law, withholding or withdrawing life-prolonging treatment is generally considered unacceptable in many Muslim societies (Al-Shahri 2016; Aramesh and Shadi 2007; Cavlak et al. 2007; Hosseinzadeh and Rafiei 2019; Weng et al. 2021). However, in Islamic law, human dignity is considered inviolable, and decisions to withhold life-sustaining treatments (LSTs), such as cardiopulmonary resuscitation, are primarily based on the anticipated futility of the intervention. Furthermore, (LSTs) may only be withdrawn if their continuation would not meaningfully contributeto the patient’s survival, with the intention being not to hasten death but to avoid futilely aiming at prolonging life. While depriving a person of vital needs such as food and water is normally considered an act of passive killing, withdrawing and/or withholding futile (and potentially harmful) artificial nutrition and hydration is considered appropriate, particularly in dying patients with far-advanced cancer (Al-Shahri 2016; Chamsi-Pasha and Albar 2017; Daar and Khitamy 2001). In some Muslim societies, the decision not to resuscitate or to withhold or withdraw other LSTs requires the agreement of at least 3 physicians and a detailed medical explanation. It is considered to be a pure medical decision (IIF-Academy 1986; Gouda et al. 2018; Islam Question & Answer 2008). While exploring all treatment options is essential for clinical decisions at the end of life (EOL), some decisions made for Muslim patients may neglect critical considerations such as the proportionality of treatment benefits, as well as patient autonomy and family preferences and cost. This can lead to interventions that may not align with the patient’s values or best interests (Almansour et al. 2019; Baharoon et al. 2010; Fearon et al. 2019). The transition from curative to PC is generally challenging for patients and their families. In Muslim populations, this difficulty may be compounded by the suboptimal explanation of the concept of PC and its absolute difference from the concept of euthanasia, which contradicts religious and cultural beliefs that emphasize the hope for a cure and the value of life-prolonging measures. Additionally, concerns about how such decisions may be perceived within their community may add another layer of complexity (Almansour et al. 2019; Fearon et al. 2019; Weng et al. 2021). The principles of PC, namely affirming life, relieving suffering, allowing natural death, and treating the dying with compassion and dignity are perfectly aligned with Islamic theology (Al-Shahri 2016). Nevertheless, a number of barriers may prevent Muslim patients from receiving PC services. These include health system issues such as a lack of resources for PC in Muslim-majority countries and a lack of culturally sensitive training for health-care professionals as well as, a lack of awareness of cultural perspectives on death in non-Muslim-majority countries. Furthermore, Muslim patients and their families may refuse PC if the aims and advantages of PC were not optimally explained to them. Challenges could also arise when PC practices appear to contradict religious expectations, for example when futile life-sustaining measures are perceived as really life-sustaining (Al-Awamer and Downar 2014; Almansour et al. 2019; Jansky et al. 2017; Weng et al. 2021).

## Review questions

The aim of this scoping review is to provide an overview of the current issues that have been studied and influence access to and use of PC, as well as the EOL decisions by people of Muslim faith in countries with and without a Muslim majority. This includes the following research questions:

- What are the preferences and practices of people of Muslim faith regarding EOL decisions in countries with and without Muslim majorities?
- How and who makes decisions or discusses EOL care by people of Muslim faith in countries with and without a Muslim majority?
- What factors and barriers have been studied that are associated with the use of PC by people of Muslim faith at the EOL in countries with and without a Muslim majority?
- What interventions have been studied to facilitate the use of PC at the EOL among people of Muslim faith in countries with and without a Muslim majority?

## Inclusion criteria

### Population

Studies involving adult Muslim patients, their families or healthy Muslim individuals, Islamic scholars or imams, and Muslim health-care professionals, Muslim students of health-care professions, or non-Muslim health-care professionals caring for Muslim patients were included. Studies that included these and other groups of participants were included if they provided data for the target group alone.

### Concept

The scoping review considered the use of PC and related barriers and facilitators, as well as preferences and decisions at the EOL, as described by Ruppert (2019). These include euthanasia (active euthanasia), “letting die” (withdrawal or withholding of one or more LSTs or medications), assisted suicide, EOL therapy, palliative sedation, and fasting to death or voluntary stopping eating and drinking.

### Context

This review included studies conducted in a treatment setting, such as a hospital, hospice, or PC unit, and studies conducted in a non-treatment setting, including long-term care facilities, community care, or other settings. Studies conducted in countries with or without a Muslim majority were also included. There was no restriction on the publication date.

### Types and language of evidence sources and publications

Qualitative and quantitative empirical studies were included, regardless of the number of participants. Study protocols and studies published only as abstracts were excluded. Studies written in English, German, and Arabic were included.

## Methods

This scoping review was conducted using the Joanna Briggs Institute (JBI) methodology (Aromataris and Munn 2020).

### Search strategy

The electronic literature search for this scoping review was conducted in the following databases: MEDLINE (via PubMed), CINAHL, The Cochrane Central Register of Controlled Trials (CENTRAL), and Web of Science. Keywords from relevant articles and Medical Subject Headings were used to develop the search strategies. The search strategies for MEDLINE (via PubMed) are listed in Online Appendix 1 and were modified as necessary for other databases. In addition, the bibliographies of the included studies were searched to identify further potentially relevant studies. The search was not restricted by language or year of publication.

### Evidence sources, screening, and selection

The study selection process was conducted in the context of the PRISMA Extension for Scoping Reviews (PRISMA-ScR) methodology (Tricco et al. 2018) and consisted of 4 stages: identification, preselection, eligibility, and inclusion. The reasons for exclusion of studies were documented in the PRISMA-ScR flow diagram. The steps of preselection, eligibility, and inclusion were carried out by 2 reviewers, namely I.A.S. and L.-M.W. In the case of disagreement regarding the inclusion or exclusion of a study, a discussion was held between the 2 reviewers. If no agreement was reached, a third reviewer, either S.F. or G.M., was consulted. When at least 2 reviewers agreed, then the study was included or excluded. The selection process is shown in a PRISMA-ScR flow diagram. For the selection process, we used Rayyan (Ouzzani et al. 2016), a web and mobile app for systematic reviews.

### Data extraction

For the data extracted from the evidence, a data extraction tool based on the “JBI data extraction tool for information on the source of the evidence characteristics and results” (Aromataris and Munn 2020) was developed and used to extract the data (see Online Appendix 2 for data extraction sheet). The following data were extracted: author, year of publication, title of study, country of study, methodology and design of the evidence, objectives of the study, population and sample size, study design, context and setting of the study, interventions (including details such as duration of intervention), individuals or groups compared, and results related to the questions of this scoping review. To test the completeness and applicability of the tool, the data extraction process was piloted for 6 studies. The piloting process was carried out by I.A.S. and L.-M.W. in a blinded manner. The data extraction process was performed by one reviewer (I.A.S.) and the extracted data were reviewed by other reviewers: 34 studies by R.M., 13 studies by S.F., and 12 studies by L-M.W. (JBI recommendations) (Aromataris and Munn 2020).

### Analysis and presentation of results

Data extraction sheets were analyzed, summarized, and coded according to the objectives of the scoping review. Results were analyzed descriptively and quantitatively. Qualitative results were analyzed using the process model of inductive category formation and deductive category application according to Mayring and Fenzl (2019) and coded using MAXQDA software (VERBI-Software 2022). A narrative summary is provided to summarize the findings in relation to the objectives and questions of the scoping review and where appropriate, presented in tables. Conclusions and recommendations for research were drawn at the end of the work.

## Results

### Search results

**Figure 1.**
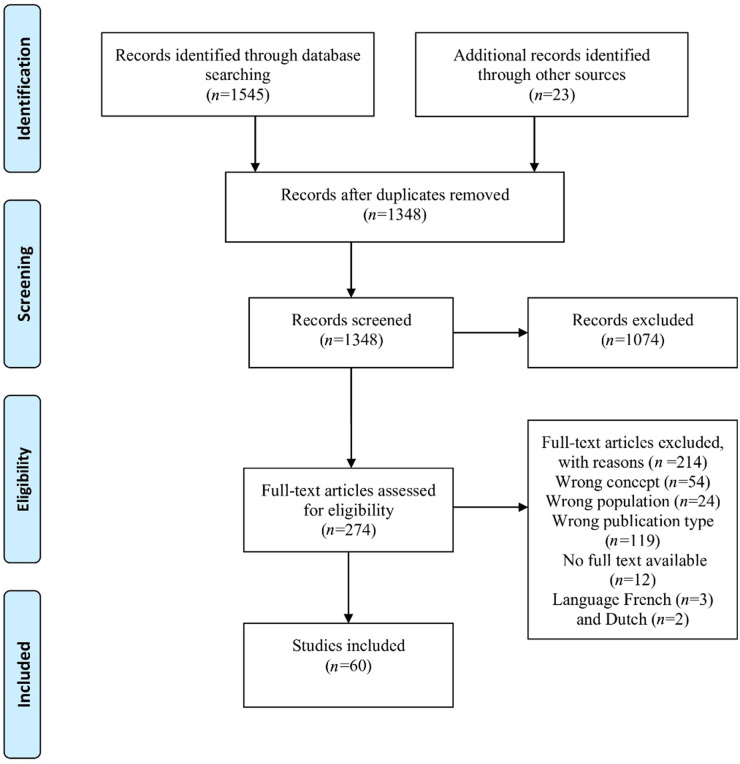
PRISMA-ScR flow diagram. PRISMA-ScR, PRISMA Extension for Scoping Reviews.

The electronic search identified 1545 articles, and 23 articles were identified through other sources (screening the reference lists of the included studies). After removing duplicates, 1348 articles remained. These were screened using the title and abstract. In the next step, 274 articles were assessed for eligibility using the full text. Of these, 60 studies met the inclusion criteria and were included in the scoping review (Abbas et al. 2021; Abudari et al. 2016; AbuYahya et al. 2021; Aghababaei and Aghababaei 2012; Ahaddour et al. 2017, 2018; Ahmed and Kheir 2006; Ahmed et al. 2001, 2010; Al-Awamer and Downar 2014; AlFayyad et al. 2019; Al-Jahdali et al. 2009; Almansour et al. 2019, 2020; Almuzaini et al. 1998; Alrimawi et al. 2017; Alsaati et al. 2019; Alshamsi et al. 2018; Alwadaei et al. 2019; Askar et al. 2000; Askarian et al. 2020; Baeke et al. 2012; Baharoon et al. 2010; Bahramnezhad et al. 2018; Bani Melhem et al. 2020; Baykara et al. 2020; Borhani et al. 2014; Cavlak et al. 2007; Colak et al. 2014; Duffy et al. 2006; Duivenbode et al. 2019; El Jawiche et al. 2020; Farid et al. 2017; Fearon et al. 2019, 2021; Gouda et al. 2018; Hammami et al. 2015, 2016; Hamouda et al. 2021; Hosseinzadeh and Rafiei 2019; Iyilikci et al. 2004; Jansky et al. 2017; Khalid et al. 2013, 2021; Muishout et al. 2018, 2022a, 2022b; Naseh and Heidari 2017; O’Neill et al. 2017; Oosterveld-Vlug et al. 2017; Ouanes et al. 2012; Ozcelik et al. 2014; Razban et al. 2016; Saeed et al. 2015; Vattanavanit et al. 2017; Weng et al. 2021; Wolenberg et al. 2013; Yildirim 2020; Zafar et al. 2016; Zamer and Volker 2013). The study selection process is shown in the PRISMA-ScR flow diagram (Figure 1).

### Review findings

#### Description of the included studies

##### Country of study

Of the 60 included studies, 43 were conducted in Muslim-majority countries (Saudi Arabia (*n* = 14), Iran (*n* = 8), Turkey (*n* = 6), Bahrain (*n* = 3), Kuwait (*n* = 2), Mauritania (*n* = 2), Sudan (*n* = 2), Jordan (*n* = 2), and one study each in Tunisia, Pakistan, Lebanon, the United Arab Emirates, and the Palestinian Territories) and 16 in non-Muslim-majority countries (the USA (*n* = 6), Belgium (*n* = 3), the Netherlands (*n* = 4), and one each in Germany, Canada, and Thailand). One study was conducted as a multinational study (an international online survey).

##### Participants

Twenty-five studies involved health-care professionals as follows: physicians (*n* = 14), nurses (*n* = 6), PC experts (*n* = 2), health-care professionals without an Islamic background (*n* = 1), and others (*n* = 2). Other studies involved students of health-care professions (nursing and medicine) (*n* = 7), patients (*n* = 9), imams (*n* = 1), and religious leaders (*n* = 1), and the remaining studies involved others.

##### Years published

Studies were published between 1998 and 2022, where most of the articles were published recently.

##### Study design

A quantitative study design was used in 36 studies, a qualitative study design was used in 17 studies, and 7 studies used a mixed-methods design. Further details are in Table 1.

**Table 1.** Description of included studies

Study-ID	Objectives	Methods	Country of study	Population and sample size
Abbas et al. 2021	Assessment of medical students’ knowledge and attitudes toward do-not-resuscitate (DNR) decisions	Cross-sectional study/online questionnaire	Saudi Arabia	425 Medical students
				Inclusion criteria: medical college students
				Exclusion criteria: first-year students and Fakeeh Medical College students of all years of study
				Female: 72.7%
				Age: (mean: 22.3) years
Abudari et al. 2016	Exploring the experiences of non-Muslim nurses who caring for terminally ill Muslim patients and their families, and the contexts that influence these experiences as described by the nurses	Qualitative descriptive through interviews, the Stevick–Colaizzi–Keen method was used for data analysis	Saudi Arabia	10 Non-Muslim nurses
				Inclusion criteria: non-Muslim nurses with at least 2 years’ experience of caring for terminally ill Muslim patients, able to communicate in English
				Exclusion criteria: Nurses with experience of being a patient in the hospital, Arab non-Muslim nurses
				Age: (range: 29−57) years
				Nationality: 3 nurses from the Philippines, 2 New Zealand, and 1 each from the UK, Canada, Ireland, India, and South Africa
				Specialties: 5 palliative care/oncology nurses, 3 oncology nurses, and 2 medical nurses
				Years of experience in caring for terminally ill Muslim patients: (2−19) years
AbuYahya et al. 2021	Assessment of the attitudes of oncology nurses toward DNR orders and the impact of religion on their attitudes	Cross-sectional study/questionnaire	Saudi Arabia	157 Nurses at a comprehensive cancer center
				Female: 91%
				Age: (mean: 34.5 ± 6.5) years
				Married: 66.7%
				Religion: Muslim 19.2%, Christian 70.5%, Hindu 8.3%, other 1.9%
				Oncology nursing experience: (mean: 7.3 ± 6) years
				Education level: Diploma 28.2%, Bachelor 68.6%, Master 3.2%
Aghababaei and Aghababaei 2012	Investigation of the relationship between religion and euthanasia, and comparison of single-item and multi-item scales of attitudes toward euthanasia	Cross-sectional study/questionnaire	Iran	300 Students from the University of Tehran
				Female: 66%
				Age: (mean: 22.6 ± 2.5) years
Ahaddour et al. 2017	Exploring the attitudes and beliefs of middle-aged and older Moroccan Muslim women living in Antwerp (Belgium) toward withholding and withdrawing curative and life-sustaining treatment	Qualitative research/grounded theory methodology using semi-structured interviews	Belgium	30 Moroccan Muslim women
				First group: 15 middle-aged Moroccan Muslim women; age: (range: 41−55) years
				Second group: 15 elderly women; age: (range: 61−86) years
	To determine whether there is a change in attitudes and beliefs between middle-aged and older Moroccan Muslim women			
	To examine the role of religion in the attitudes			
	To show how the findings relate to the existing Islamic literature			
Ahaddour et al. 2018	To investigate the relationship between contemporary normative Muslim views on assisted suicide and voluntary euthanasia on the one hand, and the actual views and attitudes of Muslims living in Belgium on the other	Qualitative research/grounded theory methodology using semi-structured interviews	Belgium	30 Moroccan Muslim women
				First group: 15 middle-aged Moroccan Muslim women; age: (range: 41−55) years
				Second group: 15 elderly women; age: (range: 61−86) years
	To determine whether there is a change in views and attitudes toward active euthanasia between first- and second-generation Muslims			
Ahmed et al. 2001	Assessment of the attitudes of Sudanese junior and senior physicians toward euthanasia and assisted suicide	Cross-sectional study/questionnaire	Sudan	248 Sudanese physicians
				Female: 48%
				Age: (mean: 38 ± 13.5) years
				Religion: Muslims: 92% with moderate adherent to Islamic teachings; Christians: 8%
				Education: graduated from Sudanese universities: 72%, the remainder graduated from different Arab and Eastern European medical school
Ahmed and Kheir 2006	Exploration of final-year medical students’ attitudes toward euthanasia and identification of factors influencing their attitudes	Cross-sectional study/questionnaire	Sudan	152 Final-year medical students (141 were included in analysis)
				Dropout: 7.2%: 11 expressed no opinion on euthanasia
				Female: 43.3%
				Age: (range: 23−27) years
				Religion: Muslim 100% described themselves as very religious 57.2% and as moderately religious 42.8%
				Number of terminally ill patients in the last 6 months: ≥3 patients 33.3%, <3 patients 66.6%
Ahmed et al. 2010	Exploring the views of people in Kuwait on the acceptability of a life-ending intervention such as physician-assisted suicide	Cross-sectional study/questionnaire	Kuwait	330 Students at the College of Social Sciences
				Female: 66.6%
				Age: (mean: 21 ± 1.7) years
				Religion: Muslim 100%
Al-Awamer and Downar 2014	Exploring the differences between palliative care (PC) models in Western countries and Muslim Middle Eastern countries in order to inform a culturally acceptable model of PC that meets the needs of Muslim Middle Eastern patients and their families	Qualitative empirical study	Canada	13 English-speaking PC experts with experience in Western and Muslim Middle Eastern countries: 1 care manager and 12 physicians (6 based in the Middle East and 6 based in the West)
				Female: 38.5%
				Religion: Muslim 100%
AlFayyad et al. 2019	Investigation of the knowledge and attitudes of physicians and nurses toward advance directives for cancer patients	Cross-sectional study/questionnaire	Saudi Arabia	281 Health-care professionals (170 nurses and 111 physicians) caring for terminally ill cancer patients
				Female: physicians 19.5%, nurses 86.5%
				Age: physicians (mean: 33.4 ± 7.6) years, nurses (mean: 33.9 ± 6.6) years
				Education level: physicians (Bachelor 63.3%, Master 11.9%, PhD 24.8%), nurses (Bachelor 93.1%, Master 6.9%, PhD 0%)
Al-Jahdali et al. 2009	Determination of resuscitation preferences in hemodialysis patients	Cross-sectional study/questionnaire	Saudi Arabia	100 Dialysis patients
				Female: 45%
				Age: (mean: 51.1 ± 15.5) years
				Nationality: Saudis 67%
				Married: 85%
				Religiosity scores: above-average 70%
				Mean duration on dialysis: (6.0 ± 4.1) years
Almansour et al. 2019	Assessment of Jordanian critical care staff perceptions of the intensity and frequency of barriers and facilitators to providing end-of-life care (EOLC)	Cross-sectional study/questionnaire	Jordan	104 Critical care staff (76 nurses and 28 physicians)
				Female: nurses 50%, physicians 45.7%
				Age (mean): nurses 26.4 ± 2.9 years, physicians 27.2 ± 0.96 years
				Education level: nurses (Bachelor 85.5%, Master 14.5 %), physicians (Bachelor 100% Master 0%)
				Years in critical care unit (mean): nurses 3.4 ± 2.0, physicians 1.3 ± 0.8
Almansour et al. 2020	Examining the characteristics, mortality rates, and treatments received in the last days of life of patients who died in intensive care units (ICUs)	Retrospective multicenter cohort study	Jordan	3885 Patients (data from health records)
				Exclusion criteria: pediatric patients younger than 18 years and patients admitted to an ICU for less than 4 h
				Female: 43.4%
				Age: (mean: 62.6 ± 18.4) years
				Multiple comorbidities: 74%
				Cancer/no cancer: 85.6%/14.4%
				Department transfer from emergency department 46.8%, wards 37.7%, other hospital 10.5%, and operation room 5.0%
Almuzaini et al. 1998	Assessment of cancer care and hospice/PC needs of cancer patients and their carers	Cross-sectional study/questionnaire	Saudi Arabia	695 Participants (136 cancer patients, 161 Informal carers and 398 health-care professionals
				Female: 37%
				Age (years): patients <25 years: 2.2%, 25–34 years: 0.7%, 35–44 years: 8.1%, 45–54 years: 31%, ≥55 years: 58%; informal carers <25 years: 1.2%, 25−34 years: 29%, 35–44 years: 62%, 45−54 years: 13%, ≥55 years: 0%; health-care professionals: <25 years: 31%, 25−34 years: 45%, 35−44 years: 21%, 45−54 years: 2.5%, ≥55 years: 0%
Alrimawi et al. 2017	Presenting the views, opinions, and positions of the Palestinian community on the concept of do-not-resuscitate for terminally ill patients	Descriptive-qualitative design using semi-structured interviews	Palestinian Community	24 Participants
				Female 54.1%
				Age: (mean: 40,6) years
				Married: 100%
				Place of residence: city 33.3%, country 33.3%, refugee camp 33.3%
				Educational level: primary education 16.6%, secondary education 41.6%, diploma 8.3%, bachelor or higher 33.3%
Alsaati et al. 2019	Assessment of medical students’ and residents’ knowledge and attitudes toward the DNR order and factors influencing their attitudes	Cross-sectional study/online survey	Saudi Arabia	429 Medical students (preclinical and clinical years)
				Female: 49.9%
				Age: (range: 20−25) years
Alshamsi et al. 2018	Investigate nephrologists’ practice of withholding and withdrawing dialysis treatment in EOL renal care for patients in a vegetative state	Cross-sectional study	United Arab Emirates	29 Nephrologists cared for patients with end-stage renal disease
				Years of professional practice: 5−10: 13.8%, 10−20: 31%, >20: 55.2%
				Profitability of dialysis unit: non-for-profit 79.3%, for-profit 20.7%
Alwadaei et al. 2019	Who should make decisions about withdrawing life-sustaining treatment	Qualitative research design using semi-structured interviews	Bahrain	12 Medical consultants (physicians with experience in the care of brain-dead patients)
	How decisions are made and the context in which decisions are made			Female: 50%
				Nationality: Bahraini citizens 100%
				At least 15 years’ experience in clinical medicine: 100%
				Medical training and experience in clinical practice outside Bahrain (mainly in Western countries): 100%
				Contact with terminally ill patients in their clinical practice and had to make and facilitate EOL decisions: 100%
Askar et al. 2000	Examine the effect of physician characteristics such as nationality, qualifications, years of experience, and/or religion on their attitudes toward euthanasia	Cross-sectional study/questionnaire	Kuwait	228 Physicians
				Female: 27.6%
				Age: (mean: 43.21 ± 9.0) years
				Nationality: Arab 40%, Kuwaiti citizens 26%, Asian 16.7%, European, American, and others 16.7%
				Religion: Muslim 72%, Christian 20%, Hindu/other 7%
				Years of professional experience: <13: 35.1%, 14−21: 32%, 22−43: 32.9%
				Place of basic medical education: Kuwait 8.3%, Arab countries 47.4%, Asian countries 16.7%, Europe, America and others 27.6%
Askarian et al. 2020	Investigating the impact of Islamic PC on breast and blood cancer patients’ quality of life	Before and after intervention study	Iran	25 Nurses working in oncology departments and 123 cancer patients receiving PC
				Inclusion criteria: hospitalization of at least 1 day (24 h), age between 18 and 60 years, willingness to participate and knowledge of their own disease, ability to report the necessary information correctly, time since diagnosis of at least 2 months
Baeke et al. 2012	To determine how migrant women engage with the popular Western right-to-die discourse and contemporary debates about active patient euthanasia	Qualitative design/grounded theory	Belgium	30 Muslim women (15 Moroccan and 15 non-Moroccan)
				Age: (range: 55−73) years
				Language spoken: Turkish: all 15 women spoke Turkish as mother tongue, the Moroccans: 8 women spoke Tarifit (a Berber language, as mother tongue) and 7 women spoke Arabic
				Knowledge of Dutch: All participants had no or very limited knowledge
				All were first-generation migrants
Baharoon et al. 2010	Identify preferences for EOLC and differences in confidence in the use of cardiopulmonary resuscitation and life-sustaining measures in the event of cardiac arrest	Cross-sectional study/questionnaire	Saudi Arabia	100 Adult patients
				Inclusion criteria: adult patients on hemodialysis for at least 2 years and not on the transplant list
				Female 46%
				Age (mean): “certainty” response group 53.3 ± 14.9 years, “uncertainty” response group 46.8 ± 16.0 years
				Married: 15%
				Nationality: Saudi citizens 67%, non-Saudi 33%
				Working status: not working 90%
Bahramnezhad et al. 2018	Exploration of the perspective of Iranian families of Muslim cancer patients on the acceptance or rejection of the DNR order	Qualitative study using content analysis	Iran	18 Participants (6 the oldest sons of the patients, 5 the husbands of the patients, 5 the fathers of the patients, and 2 daughters of the patients who were also nurses)
				Inclusion criteria: father, first son of the family, and legal guardian of terminal patients who had been told by physicians that there was no hope for them to recover their lives
				Age: (mean: 40.1 ± 12.8) years
Bani Melhem et al. 2020	Exploring advance care planning engagement among Muslims living in the USA	Cross-sectional study/questionnaire	USA	148 Muslim adults living in the United States
	Examine differences in advance care planning engagement among participants according to age, gender, health status, and experience of decision-making and end-of-life (EOL) medical treatments			Inclusion criteria: self-identified as Muslim, adults 18 years or older, and able to read, write, and comprehend English
				Female: 41.9%
				Age: (mean: 36.7 ± 13.1) years
				Born in the USA: 37.8%
				Country of origin: Asia 22.3%, Africa 7.4%, Arab countries 48%, Unites States 10.0%, other 8.7%
				Married: 65.5%
				Health status: poor 1.4%, fair 16.9%, good 35.8%, very good 31.8%, excellent 14.2%
Baykara et al. 2020	Identify ICU physicians’ attitudes toward EOL decisions for their patients and themselves	Cross-sectional study/online survey	Turkey	595 ICU physicians (with at least 95% completed answers were included in the analysis)
				Female: 54.5%
				Age: median (range) 39 (33−45) years
				Religious affiliation: believers 85.1%, undecided 5.2%, atheists 9.6%
				Primary medical specialty: anesthesiology 88.2%, internal medicine 9.9%, surgery 1.9%
				Years of experience: ≤2: 31.2%, 3−5: 25.3%, 6−10: 20.1%, >10: 23.4%
Borhani et al. 2014	Exploring intensive care nurses’ perspectives on EOLC	Descriptive qualitative design using semi-structured interviews	Iran	12 intensive care nurses from 3 ICUs in teaching hospitals, using a purposive sampling technique
Cavlak et al. 2007	Investigate and compare attitudes toward euthanasia among physiotherapists and physiotherapy students, and to investigate the effects of age, gender, and religious affiliation on participants’ acceptance of euthanasia	Cross-sectional study and comparative study/ questionnaire	Turkey	494 Participants (311 physiotherapists and 183 physiotherapy students)
				Female: physiotherapists 75.9%, physiotherapy students 57.4%
				Age (mean): physiotherapists 29.82 ± 6.28 years, students 20.85 ± 1.15 years
				Religion: physiotherapists: Muslim 96.8%, Jewish 0.3%, Christian 1.3%, Atheist 1.9%, physiotherapy students: Muslim 96.8%, Atheist 3.3%
Colak et al. 2014	Exploring the attitudes of cancer patients toward the use of morphine for pain management in a Muslim-majority country and identifying the factors that influence patients’ decisions to accept or refuse morphine for cancer pain	Cross-sectional study/questionnaire	Turkey	488 Cancer patients
				Female: 61%
				Age: (median: 54, range: 18−87) Years
				Primary tumor site: breast 44.4%, colorectal 19.8%, gastric 12.9% and lung 7:5%
				Stage of the disease: early stage 36.8%, locally advanced 26.6, metastatic disease 35%
				High school education: only 6.7%
Duffy et al. 2006	Exploring EOL preferences through 10 focus groups stratified by race/ethnicity and gender	Ten focus groups and a follow-up survey	USA	73 Participants in 10 focus groups: 5 Arab Muslim women, 4 Arab Muslim men, 9 Arab Christian women, 7 Arab Christian men, 9 Hispanic women, 9 Hispanic men, 8 black women, 8 black men, 8 white women, 8 white men
				Inclusion criteria: self-identification as Arab Muslim, Arab Christian, Hispanic, black, or white; English speaking; aged 50 and older; and no obvious unstable psychiatric or cognitive illness
				Age: (mean: 67, range: 50−83) years
Duivenbode et al. 2019	Describing the attitudes of American Muslim physicians toward EOLC decision-making and to examine the associations between physician religiosity and their attitudes	Cross-sectional study/questionnaire	USA	255 Physicians
				Female: 30%
				Age: (mean 52.1 ± 15.8) years
				Race/Ethnicity: South Asian 69.6%, Arab/Middle Eastern 21.9%
				Religious affiliation with Islam: Sunni 9.9%, Shi’ite 4.5%
				Years of medical practice: (mean: 19.1 ± 14.9)
				Practiced medicine in the USA for over 10 years 72%
El Jawiche et al. 2020	Assessment of the views and practices of intensivists in Lebanon, as well as the views of medical, legal, and religious leaders regarding the withholding of life-sustaining treatment in Lebanese ICUs	Mixed methods research: cross-sectional (survey) and qualitative design/interviews	Lebanon	83 Physicians
				Female: 39%
				Religion: Christian 68%, Muslim 22%, Druze 1%, Atheist 8%
				Specialty: critical care medicine and anesthesia 63%, critical care medicine and pulmonary medicine 34%, critical care medicine and other 3%
				Average working time in ICU: permanently: 13%, ≥50%: 36%, <50%: 51%
				Interviews: the head of the Lebanese order of physicians in Beirut, the head of the Lebanese Society of Anesthesiologists, and the head of the Lebanese Society of Critical Care Medicine, and the vice-president of the Lebanese National Consultative Committee on Ethics, a representative of the medical legal opinion in Lebanon: the lawyer of Lebanese order of physicians and representatives of the main religious confessions in the country (Christian Catholic and Orthodox, Muslim Sunni and Shia, and Druze)
Farid et al. 2017	Exploring the prediction of students’ attitudes toward euthanasia using their religious orientation, self-esteem and death anxiety	Cross-sectional study/questionnaire	Iran	247 Students (170 from the Department of Psychology and Department of Islamic Education and 77 from the Faculty of Nursing and Midwifery)
				Female: 61.1%
Fearon et al. 2019	Examine the congruence of palliative care principles with the perspectives of health-care professionals, families, and communities in rural areas of the Islamic Republic of Mauritania	Qualitative design/focus groups	Mauritania	76 Participants from across rural Mauritania (33 health-care professionals, 12 recently bereaved family members, and 31 community leaders, in 8 focus groups and 3 semi-structured interviews)
Fearon et al. 2021	Exploring the experiences of women with advanced breast cancer, their families, and health-care professionals in Mauritania	Qualitative/constructivist Stakian-multi-case study approach	Mauritania	8 Women with advanced breast cancer, 10 family members, 9 health-care professionals
				Age of women with advanced breast cancer: (range 40−70) years
Gouda et al. 2018	Exploring the possible barriers and obstacles to the practice of DNR that could improve the process of initiating a DNR order and managing patients who have been labeled as DNR	Cross-sectional study/questionnaire	Saudi Arabia	112 Physicians (71 physicians from emergency room department and 41 physicians from ICU department)
				Female: 26.8%
				Age: (mean 33.06 ± 7.90) years
	Examine the impact of Islamic religion and personal beliefs on physicians’ attitudes toward DNR and participating physicians’ advance directives			Married: 59.8%
				Religion: Muslim 97.3%, 86.6%, consider themselves reasonably religious 86.6%, non-religious 8.9%, very religious 4.5%
				Years of experience ranging from 1.0 to 23
				History of specialty training: trained in ICU/emergency room 79.5%, did not have formal specialty training in ICU/emergency room 20.5%
Hammami et al. 2015	Exploration of Saudi men’s views on EOL priorities and the usefulness of Q-methodology in this setting	Mixed methods: exploratory cross-sectional study using Q-methodology	Saudi Arabia	120 Participants (employees and patients at King Faisal Specialist Hospital and Research Centre, and companions of patients attending outpatient clinics)
				Inclusion criteria: Saudi adults 18 years or older, who had completed at least high school education, were able to understand the purpose and procedures of the study, and provided informed consent
				Age: (mean: 32.1 ± 9.8) years
				Nationality: Saudi citizens 100%
				Consider their religiosity to be the same as that of other Muslims in the Kingdom of Saudi Arabia 52%
Hammami et al. 2016	Exploring Saudi women’s EOL decisions using mean analysis and Q-methodology	Mixed methods: exploratory cross-sectional study using Q-methodology	Saudi Arabia	68 Saudi women
				Age: (mean: 30.3 ± 8.2) years
				Religiosity: much more 4%, somewhat more 31%, about the same 51%, somewhat less 9%, much less 4%
				General health status: excellent 35%, very good 43%, good 16%, fair 6%, poor 0%
				Life quality: excellent 28%, very good 51%, good 10%, fair 10%
				High school education or more 100%
Hamouda et al. 2021	Describe the perspectives and practices of American Muslim physicians regarding discussions of religion and spirituality	Cross-sectional study/questionnaire	USA	255 American Muslim physicians
				Female: 29.1%
				Age: (mean: 52 ± 15.7) years
	How physician characteristics correlate with these perspectives			Ethnicity: South Asian 70%, Arab 30%
				Sect in Islam: Sunni 95.3%, Shiite 4.5%
				Religiosity: their religion was the most or very important part of their lives 89%
				Duration in the USA: immigrated as adult 65%, immigrated as child 15.9%, born in the USA 19.2%
				Years of medical practice: (mean: 23.9 ± 15.4)
				Primary work setting: clinic/office 51.6%, hospitals 48.3%
Hosseinzadeh and Rafiei 2019	Exploring nursing students’ attitudes toward euthanasia	Cross-sectional study/ questionnaire	Iran	382 Nursing students
				Female: 61.5%
				Age: (mean: 62.6 ± 14.1) years
				Religion: Muslims 100%
				Clinical experience in hospitals during their college career: yes 51.8%
Iyilikci et al. 2004	Exploring Turkish anesthetists’ practices in withholding and withdrawing life support from critically ill patients	Cross-sectional study/questionnaire	Turkey	369 Anesthesiologists
				Females: 44.4%
				Age: (mean 40 ± 7.6) years
				Religion: 335 Muslim, 2 Jewish, 20 atheist, 12 other
				Graduation from: university hospital 78.3%, state training hospital 12.7%, social insurance training hospital 5.4%, other 35%
				Years of professional experience: ≤5: 37.7%, 6−10: 27.1%, ≥11: 35.2%
Jansky et al. 2017	Investigation of institutional requirements for the care of people with a Turkish or Arabic background in specialized PC in Lower Saxony	Mixed methods design (survey and qualitative content analysis)	Germany	55 Health-care professionals working in specialist PC
				Female: nurses 94.1%, social workers 100%, physicians 16.7%
	How often is this group of patients cared for?			Profession: physicians 13.3%, nurses 58.3%, social workers 10.0%, other professions 16.8%, no answer 1.7%
	Are there specific barriers to access to specialized PC for this patient group from the providers’ perspective?			
	What problem areas do PC professionals identify in the care of this patient group, and what are the responses and solutions?			
Khalid et al. 2013	Assessment of EOL practices among Muslim brain-dead patients, particularly with regard to the withholding and withdrawal of therapies	Quantitative/retrospective study	Saudi Arabia	42 Brain-dead patients
				Female 45.2%
				Age: (mean: 46.3 + 19.9) years
				Chronic disease in patients: none 61.9%, neoplasm 14.2%, immunosuppression, renal failure 7.1%, on dialysis 7.1%, cirrhosis 4.7%, acquired immune deficiency syndrome 4.7%
Khalid et al. 2021	Investigate the clinical characteristics, EOLC, and resuscitation status of Muslim patients who died due to COVID-19 and compare them with Muslim patients who died due to other diseases in 2020	Retrospective analysis	Saudi Arabia	96 Patients (32 in the COVID-19 group and 64 in the non-COVID group)
				Female: COVID-19 group 28%, non-COVID group 48%
				Age: (mean: COVID-19 group: 70 ± 12 years, non-COVID group: 61 ± 16 years)
				Patients with terminal disease: COVID-19 group 31%, non-COVID group 66%
Muishout et al. 2018	Exploring the professional experiences of Muslim doctors using palliative sedation in relation to religious and professional norms	Qualitative design/interpretative phenomenological study using semi-structured interviews	The Netherlands	10 Muslim physicians with professional experience in palliative sedation in general PC
				Female: 20%
				Religion: Muslim Sunni background and practicing 100%
				Specialty: 1 trainee general practitioner, 3 general practitioners, 1 trainee neurologist, 2 trainee internists, 1 geriatrician, 1 anesthetist, 1 acute medicine specialist
				Place of education and training: fully educated and trained in the Netherlands 90%
Muishout et al. 2022b	Understanding how Imams frame their role in PC decision-making/finding out what roles they see for themselves and how they construct these roles	Qualitative research design through direct content analysis/interview	The Netherlands	10 Turkish imams working in the Netherlands with experience in PC counseling
				All men
				Age: (mean 43.9 range 28−55) years
				Country of birth: 9 from Turkey, 1 from the Netherlands
				Level of education: 2 Master, 6 Bachelor, 1 post-secondary vocation education, 1 high school
				Mean years of residence in the Netherlands: 14.8 (range 3−27) years
				Years of work experience: (mean: 15.6, range 7−26)
Muishout et al. 2022a	How does the language used by Muslim physicians educated in the West and working in the West shape their attitudes and practices toward palliative decision-making	Qualitative/discourse analysis	The Netherlands	10 Physicians
				Female: 2
				Ethnic background: 7 Moroccan, 2 Turkish, 1 Afghan
				Religion: Muslim Sunni background and described themselves as religiously observant 100%
				Specialty: 1 general practitioner in training, 3 general practitioners, 1 neurologist, 2 trainee internists, 1 geriatrician, 1 anesthetist, 1 specialist in acute medicine, none of them were specialists in PC
				Professional experience in the use of palliative sedation in a general medical setting 100%
Naseh and Heidari 2017	Investigating nursing students’ attitudes toward euthanasia	Cross-sectional study/questionnaire	Iran	123 Muslim nursing students
				Dropout: incomplete questionnaires 2.4%
				Female: 65.8%
				Age: (mean 23.1 ± 1.6) years
				Married: 31.7 %
				Level of religious belief: high level 49.2%, medium level 50.8%
				Economic status: low 8.3%, middle 65.8%, high 25.8%
O’Neill et al. 2017	Describe and identify EOLC practices that nurses contribute to	Grounded theory	Bahrain	10 ICU nurses from 2 major hospitals
				Inclusion criteria: bachelor’s or associate degree holders with at least 3 years of experience working in an ICU setting
				Theoretical sampling
				Nationality: 6 Bahraini, 4 Indian
				Qualification: 4 associate degree, 6 bachelor
				Years of experience in Bahrain: range: 3.5−11
Oosterveld-Vlug et al. 2017	Investigating how people believe realistic and hopeful information should be effectively combined in physician–patient communication at the EOL	Qualitative focus group study	The Netherlands	54 Participants in 3 groups
				24 participants (patients, older people, and family members without a Muslim background) in 2 online focus groups: group A: 13 participants; female 7; age (mean: 66.1) years and group B: 11 participants; female: 4; age (mean: 82.8) years
				21 Non-Muslim health-care professionals in 2 online focus groups: group A: 10 participants; female 6; age (mean:52.0) years and group B: 11 participants; female 8; age (mean: 51.9) years
				9 Patients and carers with a Muslim background in one online focus group, female 2, age (mean: 51.7) years
Ouanes et al. 2012	Report the frequency and types of EOL decisions for dying patients in 2 ICUs, compare medical and surgical EOL practices, and identify factors associated with EOL decisions	Quantitative retrospective study design	Tunisia	326 Consecutive intensive care patients who died in the medical and surgical ICU over a period of 2 years
				Inclusion criteria: nursing student from all years of study, willingness to participate in the study
				Female 38.3%
				Age (median: 64) years
				McCabe: no fatal disease 73%, fatal disease (5 years) 20.3%, fatal disease (1 year) 6.7%
Ozcelik et al. 2014	Exploring the attitudes of undergraduate nursing students toward euthanasia	Cross-sectional study/questionnaire	Turkey	383 Nursing students
				Female: 96.9%
				Age (mean: 21.3 ± 1.5) years
				Married: 3.1%
Razban et al. 2016	Assessing critical care nurses’ attitudes to life-sustaining treatments	Cross-sectional study/questionnaire	Iran	884 ICU nurses
				Female: 92.9%
				Age: (28−35) years 58.3%
				Married: 73.8%
				Level of education: bachelor 95.2%, master 4.8%
				Years of experience in ICU: <2: 27.4%, 2−5: 36.9%, >5: 35.7%
Saeed et al. 2015	Exploring beliefs about aspects of EOLC among Muslim physicians in the USA and other countries, and assessing the impact of different factors on DNR attitudes	Cross-sectional study/online survey	Multinational (online)	461 Physicians
				Female 22.99%
				Age (years): <25: 4.2%, 25−29: 16.2%, 30−39: 40.5%, 40−49: (13.6%), >50: 25.2%
				Country of origin: South Asia 67.0%, Middle East 4.9%, USA 16.9%, others 1.7%
				Religion and sect: Sunni: 85.2%, Shia: 3.0%, Ahmadi: 0.6%
				Specialty: medical 64.86%, surgical 13.2%, critical care 1.7%, emergency room 2.8%, radiology 1.7%
				Years of experience: <5: 30.5%, 5−9: 21.4%, 10−19: 15.9%, >20: 32.1%
Vattanavanit et al. 2017	Comparing the quality of dying and palliative care in the 2 religions, Buddhism and Islam, from the perspectives of patients, families, nurses, and physicians	Mixed methods: quantitative research design/prospective survey study	Thailand	Relatives of 112 critically ill patients with chronic life-threatening conditions and acute respiratory failure (91 Buddhists and 21 Muslims) who died after admission to a medical ICU between 2015 and 2016
				Family characteristics: female: Buddhist 75.8%, Muslim 57.1%
				Age: (mean: Buddhist 44.6 ± 10.3, Muslim 42.7 ± 10.8) years
				Lived with patient: Buddhist 87.9%, Muslim 90.5%
				The nursing staff and physicians who treated them (no further details)
Weng et al. 2021	Assessing the attitudes and beliefs of health-care providers toward palliative care and the provision of palliative care services to the Bahraini community	Qualitative study/semi-structured interviews	Bahrain	16 Bahraini health-care professionals (9 physicians and 7 nurses)
				Female: physicians 5, nurses 7
	Identify the barriers that are currently preventing the development of the speciality in the region, and future steps to address them			Speciality: physicians (2 consultants, 6 residents, and 1 senior resident), nurses (1 nurse manager, 3 head nurses, and 3 nurses)
				Years of experience: <10: physicians 66.7%, nurses 28.6%, ≥10: physicians 33.3%, nurses 71.4%
Wolenberg et al. 2013	Investigate the relationship between physicians’ religious characteristics and their approaches to artificial nutrition and hydration	Cross-sectional study/questionnaire	USA	1156 Practising US physicians
				Female 35%
				Religion: Muslim 10%, Jewish 10%, Hindu 7%, Roman catholic 23%, Protestant evangelical 8%, Protestant, non-evangelical 23%, other 8%, none 12%
				Immigration history: born in the USA 63%, immigrated in the USA 37%
				Medical school training: USA medical graduate 66%, foreign medical graduate 34%
				Specialty: internal medicine 27%, family medicine 25%, cardiovascular 6%, nephrology 3%, hematology/oncology 11%, pulmonary/critical care 17%, geriatrics/hospice palliative care 12%
Yildirim 2020	Examine the knowledge, opinions, and attitudes of senior nursing students toward euthanasia and the factors that influence these attitudes	Descriptive correlation study/questionnaire	Turkey	300 Fourth-year senior nursing students
				Female: 81%
				Age (years): 21−24: 97.3%, >25: 2.7%
				Married: 1.3 %
Zafar et al. 2016	Exploring adult cancer patients’ preferences for disclosure, prognosis, and EOLC	Mixed method: (cross-sectional/qualitative method (in-depth interviews))	Pakistan	445 Cancer patients
				All patients interviewed once *n* = 420: female 58.1%, age (mean: 47.8 ± 13.9) years
				All patients interviewed twice *n* = 100: female 63%, age (mean: 44.2 ± 11.6) years
Zamer and Volker 2013	Describing the experiences of religious leaders who have supported people facing the EOL	Qualitative descriptive approach, (described by Sandelowski)	USA	4 Religious leaders from different religions (Catholicism, Judaism, Islam, and Hinduism)
				Inclusion criteria: being a religious leader, defined as an ordained person of religion and having experience in counseling people approaching the EOL
				All males
				Age: (range: 30−65) years
				Years of experience: (range: 8−36)

### Preferences and practices of people of Muslim faith regarding EOL decisions*

The majority of studies (*n* = 36) investigated preferences and practices regarding EOL decisions. These included withdrawal or withholding of one or more LSTs or medications (*n* = 30), withholding artificial nutrition or/and hydration (*n* = 5), euthanasia (*n* = 8), assisted suicide (*n* = 7), therapy at EOL (*n* = 3), and terminal and palliative sedation (*n* = 2). Voluntary stopping of eating and drinking was not reported in any of the included studies. Withdrawal of one or more LSTs or medicines was reported to be acceptable in half of the studies addressing this issue. While the results on attitudes and practices toward withholding one or more LSTs or medications present a mixed picture, they reflect varying levels of acceptance, refusal, and diverse preferences across studies. In contrast, all studies investigating euthanasia, assisted suicide, or withholding artificial nutrition and/or hydration reported negative attitudes (refusal) toward these practices. Three studies investigated physicians’ attitudes and practices regarding therapy at EOL, and 2 of them reported acceptability in this population. Similarly, 2 studies investigated terminal or palliative sedation and reported positive attitudes (acceptance) toward it. Results on one or more types of decisions at EOL possible in the same study. Table 2 provides details of EOL preferences and practices and shows the studies with negative (refusal) and positive (acceptance) attitudes toward EOL decisions described in our review.

**Table 2.** Attitudes and practices toward EOL decisions, frequency, and source study

	Acceptance (majority to absolute majority)	Refusal (majority to absolute majority)	Varied (no majority)
Withdrawal of one or more life-sustaining treatments (LSTs) or medications	*n* = 11 (Ahaddour et al. 2017; Alsaati et al. 2019; Baykara et al. 2020; Duivenbode et al. 2019; El Jawiche et al. 2020; Hamouda et al. 2021; Khalid et al. 2013; Oosterveld-Vlug et al. 2017; Razban et al. 2016; Yildirim 2020; Zafar et al. 2016)	*n* = 9 (Alshamsi et al. 2018; Askar et al. 2000; Borhani et al. 2014; Cavlak et al. 2007; Hosseinzadeh and Rafiei 2019; Iyilikci et al. 2004; Khalid et al. 2021; Muishout et al. 2022b; Ouanes et al. 2012)	*n* = 2 (Hammami et al. 2015; Ozcelik et al. 2014)
	Clarification: Baykara et al. (2020) had majority acceptance for withdrawal/withholding of one or more treatments, but not for noninvasive mechanical ventilation and enteral nutrition	Clarification: Cavlak et al. (2007) had majority refusal in both study groups (physiotherapists and physiotherapy students), but majority acceptance in the physiotherapist group	
Withholding of one or more LSTs or medications	*n* = 9 (Ahaddour et al. 2017; Baykara et al. 2020; Duivenbode et al. 2019; El Jawiche et al. 2020; Gouda et al. 2018; Khalid et al. 2013; Oosterveld-Vlug et al. 2017; Yildirim 2020; Zafar et al. 2016)	*n* = 11 (Almansour et al. 2020; Alrimawi et al. 2017; Alshamsi et al. 2018; Askar et al. 2000; Bahramnezhad et al. 2018; Cavlak et al. 2007; Hosseinzadeh and Rafiei 2019; Iyilikci et al. 2004; Khalid et al. 2021; Muishout et al. 2022b; Ouanes et al. 2012)	*n* = 6 (Abbas et al. 2021; Al-Jahdali et al. 2009; Hammami et al. 2015; Hammami et al. 2016; O’Neill et al. 2017; Ozcelik et al. 2014)
Withholding artificial nutrition or/and hydration		*n* = 5 (Almansour et al. 2020; Baykara et al. 2020; Khalid et al. 2013; Khalid et al. 2021; Wolenberg et al. 2013)	
Euthanasia (active euthanasia)	–	*n* = 8 (Ahaddour et al. 2018; Ahmed et al. 2001; Askar et al. 2000; Baeke et al. 2012; Cavlak et al. 2007; Hosseinzadeh and Rafiei 2019; Naseh and Heidari 2017; Yildirim 2020)	–
Therapy at EOL	*n* = 2 (Askar et al. 2000; El Jawiche et al. 2020)	*n* = 1 (Iyilikci et al. 2004)	–
Assisted suicide	–	*n* = 7 (Ahaddour et al. 2018; Ahmed and Kheir 2006; Ahmed et al. 2001; Ahmed et al. 2010; Askar et al. 2000; Baeke et al. 2012; Duffy et al. 2006)	–
Voluntary stopping eating and drinking	–	–	–
Palliative sedation	*n* = 2, population: physicians with experience with terminal or palliative sedation (El Jawiche et al. 2020; Muishout et al. 2018)	–	–
Advanced care planning and advanced directives	*n* = 1 (AlFayyad et al. 2019), positive attitudes toward advanced directives for cancer patients	*n* = 1 (no willingness to engage in ACP activities) (Bani Melhem et al. 2020)	–
Certainty about end-of-life care	*n* = 1, majority with certainty regarding their preferences regarding the application of cardiopulmonary resuscitation and life-sustaining measures in case of cardiac arrest (Baharoon et al. 2010)	–	–
Attitudes toward use of morphine	–	–	*n* = 1 (nearly half of the patients) (Colak et al. 2014)
Hospice/palliative care	*n* = 3 (Almuzaini et al. 1998; Duivenbode et al. 2019; Zafar et al. 2016)	–	–

### Differences and considerations related to attitudes and practices or studies with different findings on the same type of EOL decision

One study (Baykara et al. 2020) mentioned majority acceptance for withdrawal/withholding of one or more treatments, but not for fluid management, noninvasive mechanical ventilation, and enteral nutrition, another study (Ouanes et al. 2012) reported that mechanical ventilation, nutrition, and sedation were never withdrawn. Another study (Wolenberg et al. 2013) stated that Muslim physicians were significantly more likely than Catholic physicians and non-evangelical Protestant physicians to oppose withdrawing and withholding of artificial nutrition and hydration. Another study (Cavlak et al. 2007) mentioned that physiotherapists (one of the study groups) were more likely to agree with euthanasia than physiotherapy students.

### Legalization of euthanasia, assisted suicide, or withholding/withdrawal of life-sustaining treatments

Some studies reported data on whether euthanasia, assisted suicide, or withholding/withdrawal of LSTs should be legalized, and attitudes were as follows:

Regarding the legalization of euthanasia or/and assisted suicide (*n* = 5):

- Majority disagreement (refusal) (*n* = 3) (Ahmed and Kheir 2006; Ahmed et al. 2001; Askar et al. 2000).
- Varied preferences (*n* = 2) (Cavlak et al. 2007; Yildirim 2020).

Regarding the legalization of withholding or withdrawal of LSTs (*n* = 6):

- Absolute majority agreement (acceptance) (*n* = 1) (Baykara et al. 2020).
- Varied preferences (*n* = 4) (Alrimawi et al. 2017; Cavlak et al. 2007; Ozcelik et al. 2014; Yildirim 2020).
- Refusal of the legalization (*n* = 1) (Askar et al. 2000).

Another study mentioned that Muslim physicians were less agreeable for legalization than Christians and Hindus (Askar et al. 2000), and another study (El Jawiche et al. 2020) indicated opposition to euthanasia (active) by legalists from the head of the Lebanese order of physicians. In addition, one study (Alrimawi et al. 2017) stated that when legalizing the withholding of LSTs (do not resuscitate), the following points should be taken into consideration: applicable to all potential patient scenarios, each case considered separately, full responsibility of the family, protection of health-care providers, social context of the patient, age of the patient, and priority of religion.

### Factors and reasons for positive attitudes (acceptance) toward euthanasia or assisted suicide, or withholding/withdrawal of life-sustaining treatments

The following factors were identified as reasons associated with acceptance of euthanasia or assisted suicide. Further details are in Table 3:

1. Factors related to physicians and health-care professionals (including students of health professions) (*n* = 16): professional ethics or professional self-concept (*n* = 6), gender (females) (*n* = 3), having a clinical experience (*n* = 2), year of study (more acceptance in third and fourth year) (*n* = 1), atheistic viewpoint (*n* = 1), euthanasia for self (*n* = 2), high level of empathy (*n* = 1), graduates of foreign schools (*n* = 1), this study was conducted in Sudan, which means that the authors may have meant countries outside the Arab world, such as EU countries and American countries, as well as other non-Arab countries, having a Master’s degree (*n* = 1), age (30–39 years) (*n* = 1), supportive nurse behavior toward euthanasia requests (*n* = 1), and practicing of faith within the scope of science (*n* = 1).

**Table 3.** Factors and reasons for positive attitudes (acceptance) toward euthanasia, assisted suicide, or withholding/withdrawal of LSTs, frequency, and source study

Factors related to physicians and health-care professionals (including students of health professions) (*n*=16)
Professional ethics or professional self-concept (*n* = 6) (Alwadaei et al. 2019; Askar et al. 2000; El Jawiche et al. 2020; Muishout et al. 2022a; Muishout et al. 2018; Oosterveld-Vlug et al. 2017)
Gender (female) (*n* = 3) (Ahmed and Kheir 2006; Iyilikci et al. 2004; Razban et al. 2016)
Having a clinical experience (*n* = 2) (Cavlak et al. 2007; Hosseinzadeh and Rafiei 2019)
Euthanasia for self (*n* = 2) (Ozcelik et al. 2014; Yildirim 2020)
Year of study (more acceptance in third and fourth year) (*n* = 1) (Hosseinzadeh and Rafiei 2019)
Atheistic viewpoint (*n* = 1) (Baykara et al. 2020)
High level of empathy (*n* = 1) (Hamouda et al. 2021)
Graduates of foreign schools (*n* = 1) (Ahmed et al. 2001)
Having a master’s degree (*n* = 1) (Razban et al. 2016)
Age (30−39 years) (*n* = 1) (Baykara et al. 2020)
Supportive nurse behavior toward euthanasia requests (*n* = 1) (Yildirim 2020)
Practicing of faith within the scope of science (*n* = 1) (Alwadaei et al. 2019)
Principles of good death and dying (*n* = 15)
Autonomy and self-determination: the right to die (time and manner of death), belief in the right to euthanasia, and the patient’s wish and request (*n* = 9) (Ahaddour et al. 2018; Ahmed and Kheir 2006; Ahmed et al. 2010; Baeke et al. 2012; Baykara et al. 2020; Duivenbode et al. 2019; Hosseinzadeh and Rafiei 2019; Naseh and Heidari 2017; Yildirim 2020)
Quality of life at the end of life (*n* = 8) (Ahaddour et al. 2017; Alwadaei et al. 2019; Baeke et al. 2012; Duivenbode et al. 2019; Gouda et al. 2018; Muishout et al. 2022a; Oosterveld-Vlug et al. 2017; Yildirim 2020)
Helping patients to die with dignity (*n* = 2) (Ahaddour et al. 2017; Ahmed and Kheir 2006)
Ending the patient’s suffering (*n* = 2) (Ahmed and Kheir 2006; Yildirim 2020)
Reducing helplessness and hopelessness (*n* = 1) (Yildirim 2020)
life-sustaining treatments (LSTs) can be humiliating to the patient (*n* = 1) (Razban et al. 2016)
Patient-related reasons (*n* = 14)
Intolerable suffering (unbearable pain) (*n* = 4) (Ahaddour et al. 2018; Baeke et al. 2012; Hosseinzadeh and Rafiei 2019; Muishout et al. 2018)
Imminent threat of death or imminent death (*n* = 2) (Al-Jahdali et al. 2009; Khalid et al. 2021)
Type and severity of suffering (*n* = 1) (Ahmed et al. 2010)
Multiple failed resuscitations (*n* = 1) (Alrimawi et al. 2017)
Life-sustaining machines are often painful for the patient (*n* = 1) (Razban et al. 2016)
Irreversible, fatal condition (*n* = 1) (Baeke et al. 2012; Ozcelik et al. 2014)
Metastatic cancer not responding to treatment (*n* = 1) (Baykara et al. 2020)
Irreversible coma (*n* = 1) (Baeke et al. 2012)
Elderly patients over 90 years (*n* = 1) (O’Neill et al. 2017)
Related effects (old, wanting, suffering, dependent, and incurable) (*n* = 1) (Ahmed et al. 2010)
Extreme pain and total dependency (*n* = 1) (Ahmed et al. 2010)
In case of total dependence (*n* = 1) (Ahmed et al. 2010)
Confirmation of brain death (*n* = 1) (Khalid et al. 2013)
McCabe score more than 1 (*n* = 1) (Ouanes et al. 2012)
Only if bedridden, seriously ill, unable to take medication orally (*n* = 1) (Baeke et al. 2012)
Absence of a life prognosis for the patient (*n* = 1) (Alrimawi et al. 2017)
Poverty (*n* = 1) (Alrimawi et al. 2017)
Family-related factors (*n* = 8)
Burdens on the family (*n* = 3) (Ahaddour et al. 2017; Baeke et al. 2012; Naseh and Heidari 2017)
Consent of the family (*n* = 2) (Ahmed et al. 2001; Iyilikci et al. 2004)
Knowledge of health care by family members (*n* = 1) (O’Neill et al. 2017)
Application of euthanasia by relatives to unconscious patients (*n* = 1) (Yildirim 2020)
A kind of dedication (give others another chance to live) (Bahramnezhad et al. 2018)
Medical justifications (*n* = 7)
Medical futility (no benefits from these treatments) (*n* = 3) (Ahaddour et al. 2017; Al-Jahdali et al. 2009; Alwadaei et al. 2019)
Support or agreement from 2 or more physicians (*n* = 2) (Ahmed and Kheir 2006; Muishout et al. 2022b)
Approval of medical committee (*n* = 1) (Ahmed and Kheir 2006)
Availability of alternatives to curative care (e.g. hospice care) (*n* = 1) (Ahmed et al. 2001)
High annual proportion of terminally ill patients in ICU (*n* = 1) (Baykara et al. 2020)
Implementation by a committee of health-care professionals (*n* = 1) (Ozcelik et al. 2014)
Unavailability of ICU beds (*n* = 1) (Baykara et al. 2020)
The need of organ donation and transplantation (*n* = 1) (Alwadaei et al. 2019)
Religion and religious arguments (*n* = 4)
Islamic perspectives on a good death and the dying process (*n* = 2) (Muishout et al. 2018; Saeed et al. 2015)
Theological arguments (*n* = 2) (Ahaddour et al. 2017; Borhani et al. 2014)
Others (*n* = 3)
Legalization of euthanasia (*n* = 1) (Yildirim 2020)
Personality perspectives on a good death (better to take her home to see everyone and have a peaceful passing) (*n* = 1) (O’Neill et al. 2017)
Age (of healthy women, middle-aged) (*n* = 1) (Ahaddour et al. 2017)
2. Principles of good death and dying (*n* = 15): autonomy and self-determination (*n* = 9), quality of life at the EOL (*n* = 8), helping patients to die with dignity (*n* = 2), ending the patient’s suffering (*n* = 2), reducing helplessness and hopelessness (*n* = 1), and LSTs that can be humiliating to the patient (*n* = 1).
3. Patient-related reasons (*n* = 14): these include intolerable suffering (unbearable pain) (*n* = 4), imminent threat of death or imminent death (*n* = 2), type and severity of suffering (*n* = 1), multiple failed resuscitations (*n* = 1), life-sustaining machines that are often painful for the patient (*n* = 1), irreversible, fatal condition (*n* = 1), metastatic cancer not responding to treatment (*n* = 1), irreversible coma (*n* = 1), elderly patient (*n* = 1), related effects (old, patient’s request, suffering, dependent, and incurable) (*n* = 1), extreme pain and total dependency (*n* = 1), in case of total dependence (*n* = 1), confirmation of brain death (*n* = 1), McCabe score more than 1 (*n* = 1), only if bedridden, seriously ill, unable to take medication orally (*n* = 1), absence of a life prognosis for the patient (*n* = 1), and poverty (*n* = 1) (the McCabe score is a prognosis tool used to assess the severity of disease and predict patient outcomes) (McCABE and Jackson 1962).
4. Family-related factors (*n* = 8): burdens on the family (*n* = 3), consent of the family (*n* = 2), knowledge of health care by family members (*n* = 1), application of euthanasia by relatives to unconscious patients (*n* = 1), and a kind of dedication (give others another chance to live).
5. Medical justifications (*n* = 7): medical futility (no benefits from these treatments) (*n* = 3), support or agreement from 2 or more physicians (*n* = 2), approval of medical committee (*n* = 1), availability of alternatives to curative care (e.g. hospice care) (*n* = 1), high annual proportion of terminally ill patients in ICU (*n* = 1), implementation by a committee of health-care professionals (*n* = 1), unavailability of intensive care unit (ICU) beds (*n* = 1), and the need of organ donation and transplantation (*n* = 1).
6. Religion and religious arguments (*n* = 4).
7. Others: (*n* = 3): legalization of euthanasia (*n* = 1), individual perspectives on a good death (better to take her home to see everyone and have a peaceful passing) (*n* = 1), and age (of healthy women) (middle-aged) (*n* = 1).

### Factors and reasons for negative attitudes (refusal) toward euthanasia or assisted suicide, or withholding/withdrawal of life-sustaining treatments

The following factors were reported as reasons associated with refusal of euthanasia or assisted suicide and other kinds of terminal care: religion, religiosity and theological beliefs and arguments (*n* = 25), ethical and moral considerations (*n* = 12), medical and practical considerations (*n* = 10), family-related factors (*n* = 8), physician-related factors (*n* = 6), patient-related factors (*n* = 5), social and cultural factors (*n* = 5), legal concerns and regulatory issues (*n* = 4), and others (*n* = 7). Reasons for refusal are explained in Table 4.

**Table 4.** Factors and reasons for negative attitudes (refusal) toward euthanasia, assisted suicide, or withholding/withdrawal of LSTs, frequency, and source study

Religion, religiosity, and theological beliefs and arguments (*n* = 25)
Theological arguments (*n* = 16) (Ahaddour et al. 2017; Ahaddour et al. 2018; Alrimawi et al. 2017; Baeke et al. 2012; Bahramnezhad et al. 2018; Borhani et al. 2014; Fearon et al. 2019; Fearon et al. 2021; Gouda et al. 2018; Hosseinzadeh and Rafiei 2019; Muishout et al. 2022b; Muishout et al. 2018; Oosterveld-Vlug et al. 2017; Ozcelik et al. 2014; Weng et al. 2021; Yildirim 2020)
Religiosity or high religious orientation (*n* = 9) (Aghababaei and Aghababaei 2012; Ahmed and Kheir 2006; Ahmed et al. 2001; Alwadaei et al. 2019; Cavlak et al. 2007; Duffy et al. 2006; Duivenbode et al. 2019; Farid et al. 2017; Naseh and Heidari 2017)
Islamic perspectives on a good death and the dying process (*n* = 1) (Muishout et al. 2018)
Ethical and moral considerations (*n* = 12)
Ethical equivalence with suicide and murder (*n* = 4) (Ahaddour et al. 2018; Baeke et al. 2012; Oosterveld-Vlug et al. 2017; Ozcelik et al. 2014)
Euthanasia as unethical (*n* = 3) (Ahmed and Kheir 2006; Alrimawi et al. 2017; Ozcelik et al. 2014)
Professional ethics of physicians (valuing life beliefs) (*n* = 2) (Muishout et al. 2018; Naseh and Heidari 2017)
Prioritizing life extension (*n* = 2) (Naseh and Heidari 2017; Yildirim 2020)
Personal conscience and conscience issues (*n* = 2) (Cavlak et al. 2007; Yildirim 2020)
Loss of confidence in the medical profession (*n* = 1) (Hosseinzadeh and Rafiei 2019)
Inconsistency with the role of medicine (*n* = 1) (Ahmed et al. 2001)
Moral obligation (*n* = 1) (Oosterveld-Vlug et al. 2017)
Medical and practical considerations (*n* = 10)
Faith in medicine to cure disease (*n* = 3) (Ahaddour et al. 2017; Fearon et al. 2019; Oosterveld-Vlug et al. 2017)
Fear of biasing future research away from better care (*n* = 2) (Ahmed and Kheir 2006; Ahmed et al. 2001)
Dependence on future medical developments (*n* = 2) (Cavlak et al. 2007; Yildirim 2020)
Possibility of improving patient’s health (*n* = 1) (Alrimawi et al. 2017)
Presence of subtle pressure on patients, fear of dependency, or humiliation (*n* = 1) (Ahmed et al. 2001)
Possibility of misdiagnosis of brain death due to no good evidence (*n* = 1) (Alwadaei et al. 2019)
Damage to patient–nurse relationships (*n* = 1) (Hosseinzadeh and Rafiei 2019)
In the case of a tolerable pain (*n* = 1) (Ahaddour et al. 2017)
Family-related factors (*n* = 8)
Family refusal (*n* = 7) (Alwadaei et al. 2019; Bahramnezhad et al. 2018; Iyilikci et al. 2004; Muishout et al. 2022a; Muishout et al. 2018; O’Neill et al. 2017; Oosterveld-Vlug et al. 2017)
Lack of medical awareness in the family (*n* = 2) (Alwadaei et al. 2019; Muishout et al. 2018)
Family obligations to continue treatment (*n* = 1) (Alwadaei et al. 2019)
Doubts about health professional’s knowledge of treatment (*n* = 1) (Fearon et al. 2019)
Forcing of feelings (*n* = 1) (Bahramnezhad et al. 2018)
Physician-related factors (*n* = 6)
Unfamiliarity with palliative care (PC) and PC treatment possibilities (*n* = 2) (Muishout et al. 2018; Oosterveld-Vlug et al. 2017)
Physician support for false hope (*n* = 1) (Oosterveld-Vlug et al. 2017)
Factors related to the size of the medical center and the ICU (*n* = 1) (Iyilikci et al. 2004)
Unfamiliar with the concept of euthanasia (*n* = 1) (Ahmed and Kheir 2006)
Unclear or poor communication from physicians (*n* = 1) (Oosterveld-Vlug et al. 2017)
Lack of courage (*n* = 1) (Cavlak et al. 2007)
Preference for coma over euthanasia (*n* = 1) (Hosseinzadeh and Rafiei 2019)
Patient-related factors (*n* = 5)
Refusal of patient or specific request (*n* = 4) (Ahaddour et al. 2017; Bahramnezhad et al. 2018; Hammami et al. 2015; Oosterveld-Vlug et al. 2017)
Having an independent functional status prior to ICU (*n* = 1) (Ouanes et al. 2012)
A difficult farewell to family and life (*n* = 1) (Ahaddour et al. 2017)
Social and cultural factors (*n* = 5)
Clergy opposition (*n* = 2) (Bahramnezhad et al. 2018; El Jawiche et al. 2020)
Effect of community spiritual healers (*n* = 1) (Al-Awamer and Downar 2014)
Social obligation to fight disease and not to give up (*n* = 1) (Oosterveld-Vlug et al. 2017)
Local culture (*n* = 1) (Alwadaei et al. 2019)
Legal concerns and regulatory issues (*n* = 4)
Fear of potential misuse by incompetent people (*n* = 2) (Ahmed and Kheir 2006; Ahmed et al. 2001)
Legal obligations (*n* = 1) (Yildirim 2020)
Lack of laws and policies (*n* = 1) (Alwadaei et al. 2019)
Loss of mental competence (*n* = 1) (Ahmed et al. 2001)
Fear of lawsuits (*n* = 1) (Alwadaei et al. 2019)
Fear of noncompliance (*n* = 1) (Ahmed et al. 2001)
Euthanasia as illegal (*n* = 1) (Yildirim 2020)
Others (*n* = 6)
Age (older) (*n* = 2) (Ahaddour et al. 2017; Ozcelik et al. 2014) (immigrants women and nursing students, respectively)
Individual definition of a good death (doing everything possible) (*n* = 1) (Fearon et al. 2019)
Personal extrinsic orientation/ university students (*n* = 1) (Aghababaei and Aghababaei 2012)
Gender (female) (*n* = 1) (Naseh and Heidari 2017)
Age (younger nursing students) (*n* = 1) (Naseh and Heidari 2017)
Arabic ethnicity (*n* = 1) (Hamouda et al. 2021)
Study year (negative correlation) (*n* = 1) (Ozcelik et al. 2014)

### Factors with no significant or specified association with euthanasia/assisted suicide, or withholding/withdrawal of life-sustaining treatments

The following factors were reported to be correlated with EOL care decisions, but the tendency of the correlation was not reported: principles of a good death and quality of life (*n* = 3) (Alrimawi et al. 2017; Alsaati et al. 2019; Baykara et al. 2020), cultural differences (*n* = 3) (Al-Awamer and Downar 2014; Alshamsi et al. 2018; Zamer and Volker 2013), treatment costs (*n* = 2) (Alshamsi et al. 2018), religion and religious beliefs (*n* = 2) (Alsaati et al. 2019; Alshamsi et al. 2018), hospital policy (*n* = 1) (Alshamsi et al. 2018), access to PC (*n* = 1) (Alshamsi et al. 2018), limited ICU space (*n* = 1) (Alsaati et al. 2019), medical considerations (risk of vegetative state (*n* = 1) (Alsaati et al. 2019); unavailability of ICU beds, disease prognosis, drug addiction, and comorbid illness (*n* = 1) (Baykara et al. 2020)), patient and family understanding of the concept of brain death (*n* = 1) (Alwadaei et al. 2019), patient’s age (*n* = 1) (Baykara et al. 2020), economic situations of family (*n* = 1) (Alrimawi et al. 2017), and legal concerns (*n* = 1) (Alsaati et al. 2019).

Other studies reported that the following factors and reasons showed no significant association or were without association: gender (*n* = 5) (Aghababaei and Aghababaei 2012; Ahmed et al. 2010; Al-Jahdali et al. 2009; Baykara et al. 2020; Cavlak et al. 2007), age (*n* = 4) (AbuYahya et al. 2021; Aghababaei and Aghababaei 2012; Al-Jahdali et al. 2009; Cavlak et al. 2007), religion and religious beliefs (*n* = 3) (AbuYahya et al. 2021; Al-Jahdali et al. 2009; Baykara et al. 2020), medical considerations (dialysis duration (*n* = 1) (Al-Jahdali et al. 2009); incurability (*n* = 1) (Ahmed et al. 2010)), health-care professionals related factors (job role and experiences (*n* = 2) (AbuYahya et al. 2021; Baykara et al. 2020), educational level (*n* = 1) (AbuYahya et al. 2021), and knowledge of Arabic language (*n* = 1) (AbuYahya et al. 2021)), economic status of patient/family (*n* = 1) (Naseh and Heidari 2017), principles of a good death and quality of life (*n* = 1) (Al-Jahdali et al. 2009), and demographic factors (marital status (*n* = 3) (AbuYahya et al. 2021; Al-Jahdali et al. 2009; Naseh and Heidari 2017), working status (*n* = 1) (Al-Jahdali et al. 2009), family size (*n* = 1) (Al-Jahdali et al. 2009), and self-esteem and death anxiety (*n* = 1) (Farid et al. 2017)).

### Confidence in making EOL care decisions and possible influencing factors

One study (Baharoon et al. 2010) investigated differences in certainty about the use of life support and life-sustaining measures in the event of cardiac arrest and possible influencing factors. This study found that the majority of participants were certain about their preferences and only 2 factors, younger age and having more than 5 children, were associated with a positive or negative effect, respectively.

### Hospice and PC (use and preferences)

Eight studies reported findings on hospice and PC (use and preferences). Three studies (Almuzaini et al. 1998; Duivenbode et al. 2019; Zafar et al. 2016) reported preferences for palliative/hospice, and all of them reported majority acceptance of recommending or continuing care in a hospice or PC unit. One of these studies (Duivenbode et al. 2019) found that physicians who had read books on Islamic bioethics were less likely to recommend hospice care. Characteristics of patients who should receive PC were described in another study (Weng et al. 2021) and included: patients in a vegetative state, no further curative options, poor prognosis, patients with a terminal diagnosis, impaired cognition, older age, and terminally ill. Three studies elaborated on limited use (Jansky et al. 2017; Khalid et al. 2021) and limited provision (Fearon et al. 2019) of PC services in this population. Another study (Vattanavanit et al. 2017) reported that PC received, symptomatic treatment, pain control, and spiritual care were more likely to be received by Muslim patients compared to other patients (both without significant effect). Preferences regarding PC preferred to be received were listed in one study (Zafar et al. 2016), and the following preferences were reported by a majority to absolute majority of participants: spiritual and religious well-being (most important consideration), psychological counseling for emotional problems, and adequate pain control and symptom management. One study (Jansky et al. 2017) identified the following reasons for admission to the PC unit: pain management, optimizing care networks, need for psychosocial support, lack of home care, and medical and nursing difficulties.

### Factors influencing patient acceptance of opioid analgesic treatment

Reasons for refusing morphine were identified in 2 studies: one study (Colak et al. 2014) highlighted religious beliefs and the desire to save morphine for later use, while fear of addiction was identified as a reason in both studies (Abudari et al. 2016; Colak et al. 2014).

### Advanced care planning engagement and influencing factors and attitudes toward advance directives

One study (Bani Melhem et al. 2020) examined engagement in advanced care planning (ACP) activities and reported that the majority of participants were not willing to engage in ACP activities and that the following factors were associated with a positive effect on engagement in ACP activities: knowledge of a person’s stated EOL preferences, knowledge of a deceased person who received aggressive or minimal treatment, experience with decision-making or EOL treatment, major surgery, experience with illness (bad/good health and serious illness), and awareness of ACP. Another study (AlFayyad et al. 2019) reported on physicians’ and nurses’ knowledge and attitudes toward advance directives for cancer patients and found positive attitudes, with the following factors being associated with positive attitudes: female gender and higher educational level (Masters and Ph.D.) and having more knowledge about advance directives, while the belief that advance directives reduce patients’ sense of hope was associated with negative attitudes.

### Decision-making and decision-maker

#### Decision-making model

The preferred decision-making model was reported in 5 studies as follows: multidisciplinary decision (absolute majority) (El Jawiche et al. 2020), shared decision-making (absolute majority) (Alshamsi et al. 2018), physician paternalism no metrics (Muishout et al. 2022a), multidisciplinary decision no metrics (Zamer and Volker 2013), shared decision, and consumerism with no clear conclusion on which model was preferred (Hamouda et al. 2021).

#### Considerations within the decision-making process

Identified considerations included consultation with 2 physicians and 1 Muslim scholar (Zamer and Volker 2013), strong preference for a Muslim physician in EOL decision-making (Muishout et al. 2022b), emphasis on providing complete and clear information (El Jawiche et al. 2020), the need for a signature from either the patient, surrogate, or family member (El Jawiche et al. 2020), consequences for physicians (Alwadaei et al. 2019), and the of support from an ethics committee for the majority (Iyilikci et al. 2004). One study (Fearon et al. 2019) mentioned that the following topics were not considered: the views of patients and families, costs, and likely benefits. Another study stated that making one’s own medical decisions was not a priority for respondents (Hammami et al. 2015).

#### Decision-maker (practice)

Findings on who was involved in the decision-making process were reported in 10 studies, but only 4 studies provided metric data on this, of which 2 studies (Iyilikci et al. 2004; Khalid et al. 2021) involved physicians in the absolute majority of cases. In the remaining 2 studies, the family was involved in the absolute majority of cases in one study (Khalid et al. 2013), and the patients’ children were the main decision-makers in half of the cases in the other study (Khalid et al. 2021). Another study (Gouda et al. 2018) reported that families and patients were not involved in the majority of cases. Other studies that did not report metrics on this topic and were conducted in Muslim-majority Middle Eastern (MME) countries reported the following involvements: nurses indirectly involved (O’Neill et al. 2017), nurses had no active role (Alrimawi et al. 2017; O’Neill et al. 2017), patients and families not involved (Borhani et al. 2014), families (main decision-making unit in MME countries) (Al-Awamer and Downar 2014), family (limited involvement) (O’Neill et al. 2017), and family “without any real involvement of the patient” (Abudari et al. 2016). In addition, one of these studies reported that the social worker had no involvement and the hospital Muslim chaplain had a minimal role (Khalid et al. 2013).

#### Who should decide or be involved in the decision-making process

Ten studies reported on who should decide or be involved in the decision-making process and showed the following results.

##### Physicians and nurses (n = 10)

Physicians should decide or be involved in the decision-making process for the majority or absolute majority of participants in (*n* = 7) studies (Al-Jahdali et al. 2009; Alrimawi et al. 2017; Alsaati et al. 2019; Askar et al. 2000; Baykara et al. 2020; El Jawiche et al. 2020; Gouda et al. 2018), physicians for a significant part (*n* = 1) (Abbas et al. 2021), one of which stated that 3 physicians were needed to make this decision by an absolute majority (Gouda et al. 2018), while another one stated that more than one “trusted” physician was needed for the majority participants (Alsaati et al. 2019). Two other studies (Alwadaei et al. 2019; Hammami et al. 2015) stated that physicians should decide or be involved, but these did not include metrics on this. One study (Alrimawi et al. 2017) reported that nurses could play a role in the decision-making process (no metrics reported on this), and another study (El Jawiche et al. 2020) mentioned that nurses should be involved for the absolute majority of respondents.

##### Family and relatives (n = 8)

Family and relatives should be involved for the absolute majority (*n* = 2) (Alshamsi et al. 2018; El Jawiche et al. 2020), and for a significant part (*n* = 2) (Askar et al. 2000; Baykara et al. 2020), family (portion not stated) in 2 other studies (Alwadaei et al. 2019; Hammami et al. 2016), and first relative in case of coma (portion not stated) in one other study (Hammami et al. 2015). Another study (Alrimawi et al. 2017) stated that the family did not have the right of choice in the decision-making process (portion not specified).

##### Patients (n = 4)

Patients should be involved in the decision-making process in 4 studies as following: patient for absolute majority (*n* = 1) (Alsaati et al. 2019), patient or their legal representatives for two-thirds majority (*n* = 1) (Baykara et al. 2020), for nearly half (*n* = 1) (Abbas et al. 2021), and patient portion not stated (*n* = 1) (Alwadaei et al. 2019).

##### Others (n = 5)

Others should decide or be involved in the decision-making process and these are listed in Table 5.

**Table 5.** Others should be involved in the decision-making process

Other types	Frequency and source study
An ethics committee (significant portion)	1 (Baykara et al. 2020)
An ethics committee (majority)	1 (El Jawiche et al. 2020)
Religious authorities (portion not stated)	1 (Alwadaei et al. 2019)
Other secular higher authorities (portion not stated)	1 (Alwadaei et al. 2019)
Society (portion not stated)	1 (Alwadaei et al. 2019)
Consensus of different participants (majority)	1 (Iyilikci et al. 2004)
A religious advisor (significant portion)	1 (Askar et al. 2000)

### Facilitators and barriers associated with the use of PC

#### Facilitators

Facilitators to support the use of PC services in the review population were reported in 9 studies and can be categorized into 10 main groups as follows: facilitators at hospital level (*n* = 7), organizational strategies at a regional/national level (*n* = 4), facilitators related to patients and their families (*n* = 4), facilitators related to health-care professionals (*n* = 3), facilitators related to communication and interaction between patients/families and health-care professionals (*n* = 3), integration of religious and cultural practices in PC (*n* = 2), PC education and training and research (*n* = 2), societal facilitators (*n* = 1), effective pain management in PC (*n* = 1), and enhancing patient-centered care/respecting patient preferences (*n* = 1). See Table 6.

**Table 6.** Facilitators associated with the use of palliative care (PC), frequency, and source study

Facilitators at hospital level (*n* = 7)
Explaining opioid therapy to families by PC physicians (*n* = 1) (Abudari et al. 2016)
Collaborating within health-care team (*n* = 1) (Borhani et al. 2014)
Ensuring availability of fully equipped hospice care support (*n* = 1) (Almuzaini et al. 1998)
Enhancing psychosocial support programs (*n* = 1) (Al-Awamer and Downar 2014)
Engaging nurses/other health teams in building the PC services (*n* = 1) (Al-Awamer and Downar 2014)
Prioritizing continuity of care for dying patients (*n* = 1) (Al-Awamer and Downar 2014)
Building relationships within health-care team (*n* = 1) (Al-Awamer and Downar 2014)
Getting administration support (*n* = 1) (Al-Awamer and Downar 2014)
Choosing the “right” personnel (*n* = 1) (Al-Awamer and Downar 2014)
Promote positive attitudes toward PC among health (*n* = 1) (Almuzaini et al. 1998)
Supporting non-licensed staff to assist in the care of dying patient (*n* = 1) (Almansour et al. 2019)
Talking with the patient about his feelings and thoughts about (*n* = 1) (Almansour et al. 2019)
Allowing families unlimited access to the dying patient (*n* = 1) (Almansour et al. 2019)
Having an ethics committee member (nurses) (*n* = 1) (Almansour et al. 2019)
Availability of professional opinion of 3 specialized reliable physicians (El Jawiche et al. 2020)
Having the physicians agree about the direction care (*n* = 1) (Almansour et al. 2019)
Allowing continuity of care for the dying patient by the same nurse (*n* = 1) (Almansour et al. 2019)
Effective pain management in PC (*n* = 1) (Jansky et al. 2017)
Organizational strategies at a regional/national level (*n* = 4)
Develop or have national PC health policies and guidelines (*n* = 2) (Al-Awamer and Downar 2014; El Jawiche et al. 2020)
Enhancing psychosocial support programs within hospitals (*n* = 1) (Oosterveld-Vlug et al. 2017)
Improve access to PC medication and services (*n* = 1) (Al-Awamer and Downar 2014)
Integrate PC programs across a region/nation (*n* = 1) (Al-Awamer and Downar 2014)
Establishment of a hospice, fully equipped with specialist staff (*n* = 1) (Almuzaini et al. 1998)
Facilitators related to patients and their families (*n* = 4)
Facilitating discussions with all parties or via a family spokesperson (*n* = 1) (Jansky et al. 2017)
Permission from family to “stop life support” (*n* = 1) (Borhani et al. 2014)
Acceptance of the reality of terminal illness among family members (*n* = 2) (Abudari et al. 2016; Almansour et al. 2019)
Allowing the family to help physically care for the dying patient (*n* = 1) (Almansour et al. 2019)
Facilitators related to health-care professionals (*n* = 3)
Emphasize respect for humanity and dignity (*n* = 2) (Borhani et al. 2014; Oosterveld-Vlug et al. 2017)
Having a good communication skills (*n* = 2) (Fearon et al. 2019; Oosterveld-Vlug et al. 2017)
Professional obligation to provide care until the last moment of the patient’s life (*n* = 1) (Borhani et al. 2014)
Having emotional relationships with patients (*n* = 1) (Borhani et al. 2014)
Facilitators related to communication and interaction between patients/families and health-care professionals (*n* = 3)
Recognition of end-of-life phase by an external person (physicians) (*n* = 1) (Fearon et al. 2019)
Existing of consent signed by family and physicians (*n* = 1) (El Jawiche et al. 2020)
A pragmatic approach to conflict resolution strategies (*n* = 1) (Jansky et al. 2017)
Offering dialogue to resolve conflicts (*n* = 1) (Jansky et al. 2017)
Provision of information material in foreign languages (*n* = 1) (Jansky et al. 2017)
Building trust (*n* = 1) (Jansky et al. 2017)
Repeated dialogue about the patient’s situation and therapy goals (*n* = 1) (Jansky et al. 2017)
Communication using picture boards or books (*n* = 1) (Jansky et al. 2017)
Providing access to translators (*n* = 1) (Jansky et al. 2017)
Integration of religious and cultural practices in PC (*n* = 2)
Involving a Muslim spiritual counselor to facilitate clarity in clarity in communication (*n* = 1) (Oosterveld-Vlug et al. 2017)
Existence of religious guidelines by Sunni Muslims (*n* = 1) (El Jawiche et al. 2020)
PC education and training and research (*n* = 2)
Expanding PC training programs for medical students and health-care professionals (*n* = 2) (Al-Awamer and Downar 2014; Jansky et al. 2017)
Educating medical staff about PC principles and practices (*n* = 1) (Al-Awamer and Downar 2014)
Training and material for the care of people with a migration background (*n* = 1) (Jansky et al. 2017)
Supporting PC research (*n* = 1) (Al-Awamer and Downar 2014)
Enhancing patient-centered care/respecting patient preferences (*n* = 1) (Jansky et al. 2017)
Acceptance of differences and recognition of each patient as an individual
Assignment of a same-sex health-care provider
Optimization of care (enabling people to perform religious rituals such as washing and praying)
Societal facilitators (*n* = 1) (Al-Awamer and Downar 2014)
Involving community and religious leaders in PC advocacy
Improving public awareness

#### Barriers

Thirteen studies reported on barriers to using PC services, and these can be sorted into 10 main categories as follows: barriers related to patients and families and their behavior (*n* = 10), laws and policies (*n* = 8), lack of education, knowledge, and exposure (*n* = 7), structure of the health-care system (*n* = 6), barriers related to cultural norms and values (*n* = 7), barriers to communication and interaction between patients, relatives, and health-care professionals (*n* = 6), lack of necessary resources (*n* = 4), barriers related to behavior of health-care professionals (*n* = 6), social pressure (*n* = 2), and religious beliefs (*n* = 2). See Table 7.

**Table 7.** Barriers associated with the use of palliative care (PC), source study, and frequency

Barriers related to patients and families and their behavior (*n* = 10)
Lack of information and awareness about the roles and benefits of PC (*n* = 3) (Almansour et al. 2019; Almuzaini et al. 1998; Jansky et al. 2017)
Rejection of family (Al-Awamer and Downar 2014) (*n* = 3) (Almansour et al. 2019; Jansky et al. 2017; Muishout et al. 2018)
Intra-family conflicts on the direction of therapies (*n* = 2) (Almansour et al. 2019; Jansky et al. 2017)
Aggressive behaviors and dealing with angry or distraught family members (*n* = 2) (Almansour et al. 2019; Jansky et al. 2017)
Problematic symptoms or psychosocial situation (*n* = 2) (Almansour et al. 2019; Jansky et al. 2017)
Reluctance to report pain (*n* = 1) (Colak et al. 2014)
Unwillingness to use opioids due to “myths about opioids” (*n* = 1) (Colak et al. 2014)
Mistrust of outsiders (health-care providers) (*n* = 1) (Colak et al. 2014)
Difficulties discussing with patients and families (*n* = 1) (Weng et al. 2021)
Financial status of the family (*n* = 1) (Fearon et al. 2019)
Rejection of care in general or specific forms from care services (resistance to use PC) (*n* = 1) (Jansky et al. 2017)
Isolation of families with a migration background (*n* = 1) (Jansky et al. 2017)
Potential existence, social network that takes care of them (*n* = 1) (Jansky et al. 2017)
Use of alternative structures (specialized care service for migrants) (*n* = 1) (El Jawiche et al. 2020)
Families’ unrealistically high expectations (*n* = 1) (Borhani et al. 2014)
Systems of families (many family members are obliged to visit (*n* = 1) (Jansky et al. 2017)
The family is not present with the dying patient (*n* = 1) (Almansour et al. 2019)
Continuous calls to physicians from family members (*n* = 1) (Almansour et al. 2019)
Interrupting work routines and disrupting the physical comfort of patients because of a religious obligation (non-Muslim nurses) (*n* = 1) (Abudari et al. 2016)
Laws and policies (*n* = 8)
Legal concerns and restrictions (*n* = 6) (Al-Awamer and Downar 2014; Almansour et al. 2019; Borhani et al. 2014; Colak et al. 2014; El Jawiche et al. 2020; Weng et al. 2021)
Absence of laws, policies, regulatory frameworks, and guidelines (*n* = 6) (Al-Awamer and Downar 2014; Almansour et al. 2019; Alwadaei et al. 2019; Borhani et al. 2014; El Jawiche et al. 2020; Weng et al. 2021)
Challenges in implementation of PC policies (*n* = 2) (Al-Awamer and Downar 2014; Colak et al. 2014)
No financial benefit in the governmental or private sector (*n* = 1) (Almuzaini et al. 1998)
Lack of education, knowledge, and exposure (*n* = 7)
Lack of familiarity with the role and benefits of PC (*n* = 2) (Al-Awamer and Downar 2014; Jansky et al. 2017)
Lack of knowledge and training in pain management (*n* = 2) (Borhani et al. 2014; Colak et al. 2014)
Limited exposure of medical trainees to PC and end-of-life (EOL) issues (*n* = 1) (Al-Awamer and Downar 2014)
Limited practical experience (*n* = 1) (Fearon et al. 2019)
Lack of education and training for clinicians (*n* = 1) (Almansour et al. 2019)
Lack of education in society (*n* = 1) (Weng et al. 2021)
Structure of the health-care system (*n* = 6)
Access problems (*n* = 3) (Al-Awamer and Downar 2014; Fearon et al. 2019; Jansky et al. 2017)
Continuing of treatment due to financial benefits to the hospital (*n* = 2) (Al-Awamer and Downar 2014; Almansour et al. 2019)
Limited availability of hospice care (*n* = 2) (Al-Awamer and Downar 2014; Weng et al. 2021)
Lack of a national integrated and accessible PC service (*n* = 1) (Al-Awamer and Downar 2014)
Lack of support programs for PC professionals (*n* = 1) (Al-Awamer and Downar 2014)
Hierarchy within the administration and within medical team (*n* = 1) (Al-Awamer and Downar 2014)
Limited integration of services across care settings (*n* = 1) (Al-Awamer and Downar 2014)
Unsafe environment (*n* = 1) (Jansky et al. 2017)
Problems by organization and planning of care (care provider from other genders) (*n* = 1) (Jansky et al. 2017)
Poor unit design that does not allow privacy for dying patients (*n* = 1) (Almansour et al. 2019)
Lack of government support for PC programs (*n* = 1) (Al-Awamer and Downar 2014)
Lack of administrative support for PC at hospital level (*n* = 1) (Al-Awamer and Downar 2014)
Lack of support for family (social worker/religious leader) (*n* = 1) (Almansour et al. 2019)
Uncomfortable environment for Muslim women (*n* = 1) (Duffy et al. 2006)
Use of alternative structures (specialized migrant care services) (*n* = 1) (Jansky et al. 2017)
Problems with visiting hours (too restrictive or too generous) (*n* = 1) (Almansour et al. 2019)
Barriers related to cultural norms and values (*n* = 7)
Dealing with the cultural differences (*n* = 4) (Abudari et al. 2016; Almansour et al. 2019; El Jawiche et al. 2020; Jansky et al. 2017)
Cultural barriers related to EOL care (*n* = 2) (Duffy et al. 2006; Jansky et al. 2017)
No acceptance of the loss of patient (*n* = 1) (Weng et al. 2021)
Truth-telling blocked by family (*n* = 1) (Al-Awamer and Downar 2014)
Barriers to communication and interaction between patients, relatives, and health-care professionals (*n* = 6)
Communication barriers in/during medical consultations (*n* = 1) (Fearon et al. 2019)
Inability to communicate with others due to coma (*n* = 1) (Borhani et al. 2014)
No space for questions (*n* = 1) (Fearon et al. 2019)
Giving a little or incorrect information (*n* = 1) (Fearon et al. 2019)
Lack or limited cognitive ability of the patient (*n* = 1) (Jansky et al. 2017)
Lack of language skills when interacting with patient and family (*n* = 1) (Jansky et al. 2017)
Different gender understanding (*n* = 1) (Jansky et al. 2017)
Problems in making contact (*n* = 1) (Jansky et al. 2017)
Language barriers (*n* = 2) (Abudari et al. 2016; Jansky et al. 2017)
Avoiding conversations with family (*n* = 1) (Almansour et al. 2019)
Unclear or poor communication from physicians (*n* = 1) (Oosterveld-Vlug et al. 2017)
Barriers related to behavior of health-care professionals (*n* = 6)
Noninvolvement of PC team due to lack of referral (*n* = 1) (Abudari et al. 2016)
Ethical conflicts due to different wishes of team/patient and family (*n* = 1) (Jansky et al. 2017)
Clinicians overly optimistic about patient survival (*n* = 1) (Almansour et al. 2019)
Clinicians who focusing on saving the patient’s life (not the quality of EOL) (*n* = 1) (Almansour et al. 2019)
Clinicians who won’t allow the patient to die (*n* = 1) (Almansour et al. 2019)
Clinicians know about the patient’s poor prognosis before family (*n* = 1) (Almansour et al. 2019)
Do not seek other opinions from other health-care professionals (*n* = 1) (Almansour et al. 2019)
Providing LST requested by family, despite patient’s signed advanced directive (*n* = 1) (Almansour et al. 2019)
Not knowing or seeking the patient’s wishes (*n* = 1) (Almansour et al. 2019)
Continuing treatment of dying patient, despite pain and discomfort (*n* = 1) (Almansour et al. 2019)
Avoiding discussion with family (*n* = 1) (Almansour et al. 2019)
Misperception about health/dealing with morbidity/mortality (*n* = 1) (Colak et al. 2014)
Resistance of medical staff to PC and late referral (*n* = 1) (Al-Awamer and Downar 2014)
Physicians not interested in working in this area (*n* = 1) (Almuzaini et al. 1998)
Disagreement between multiple clinicians about direction of care (*n* = 1) (Almansour et al. 2019)
Lack of necessary resources (*n* = 4)
Lack of qualified medical staff (*n* = 3) (Al-Awamer and Downar 2014; Borhani et al. 2014; Weng et al. 2021)
Limited availability of medication (opioids) (*n* = 2) (Al-Awamer and Downar 2014; Colak et al. 2014)
Limitations in establishing a functional multidisciplinary team (*n* = 1) (Al-Awamer and Downar 2014)
Limited support for PC research (*n* = 1) (Al-Awamer and Downar 2014)
Limited financial resources (*n* = 1) (Al-Awamer and Downar 2014)
Lack of integrated patient-centered spiritual care (*n* = 1) (Al-Awamer and Downar 2014)
Limited psychosocial support (*n* = 1) (Al-Awamer and Downar 2014)
Social pressure (fear of societal judgment and stigma) (*n* = 2) (Fearon et al. 2019; Weng et al. 2021)
Religious beliefs (*n* = 2) (Borhani et al. 2014; Weng et al. 2021)

### Interventions studied in relation to the use of PC at EOL

This was reported in one pre- and post-intervention study (Askarian et al. 2020), the intervention studied was training nurses on PC with an Islamic approach to reducing pain in cancer patients. The intervention was significantly associated with a reduction of the inadequate situation of patients, an improvement in values of quality of life, an improvement in mental health, an improvement in environmental health, and an improvement in physical health. However, an associated improvement in social health was shown to be insignificant.

## Discussion

We collected and analyzed the published literature on the use of PC services and attitudes and decisions at the EOL in Muslim populations. We identified 5 topics studied, the first one deals with preferences and decisions at the EOL, which were mostly negative (refusal) toward euthanasia and assisted suicide and withholding of one or more LSTs or medications, while there was relatively more acceptance of withdrawal of one or more LSTs or medications, therapy at EOL and palliative sedation, which may be explained that studies reporting on this topic involved physicians. The second focuses on the reasons for accepting or rejecting some of these decisions. Interestingly, our review found that while some participants in the included studies indicated a willingness to make decisions in favor of euthanasia, assisted suicide, and other decisions at the EOL because of religion and religious beliefs, this was the most common reason for refusing these types of decisions. The same was true for reasoning with principles of good death and dying. While these were reasons for accepting or agreeing with some of these decisions for some participants, they were also the reasons for refusing these decisions. Several factors could explain these inconsistent results: (a) individual differences between participants in understanding and reflecting on these issues; (b) participants’ ignorance of the meaning and definition of some of these decisions and of the existence of some religious judgments that regulate these types of EOL decisions in different scenarios; and (c) the presence of participants from groups with different professional and cultural backgrounds. The third is concerned with decision-making and decision-makers, there was a lack of clarity in a number of studies about the role of the patient in the decision-making process, in addition to a lack of studies focusing on patterns and models of decision-making. Furthermore, some studies did not have quantitative data on the decision-maker, so we were unable to make conclusive findings about this. The fourth topic shows that, despite the growing need for PC, the results of our scoping review show that there is still a vacuum in the provision of PC for Muslim patients. This may be explained by the existence of the various barriers identified in our review. The fifth topic deals with the facilitators and barriers associated with the use of PC by the Muslim population, some of which could be avoided in order to increase the use of PC by this population. The barriers reported in Muslim-majority countries tended to be in the topics of legislation, policy, lack of education, knowledge and exposure, and lack of necessary resources (Al-Awamer and Downar 2014; Almansour et al. 2019; Almuzaini et al. 1998; Alwadaei et al. 2019; Borhani et al. 2014; Colak et al. 2014; El Jawiche et al. 2020; Fearon et al. 2019; Weng et al. 2021). In non-Muslim-majority countries, they tended to be in the topic of barriers to communication and interaction between patients, relatives, and health-care professionals (Jansky et al. 2017; Oosterveld-Vlug et al. 2017). Our review did not identify any articles that examined the effect of the likely facilitators of PC use in the review population, but we also identified the facilitators of potentially increased PC use.

### Strengths and limitations

Our scoping review provides a comprehensive summary and synthesis of the findings of different studies in order to draw conclusions about the existing literature and to identify research gaps for further investigation. It includes a wide range of literature sources and types to provide a detailed understanding of the research area, and it presents the findings of the review in a clear and concise manner to improve readability and interpretability. To the best of our knowledge, this scoping review is the first looked at barriers and facilitators to PC use and preferences among Muslims in both Muslim-majority and non-Muslim-majority countries. This not only addresses an existing gap in knowledge but also highlights the importance of this review. The literature search was conducted in 4 databases (due to time and resource constraints) and was limited to certain languages or publication types; these limitations may result in missing relevant studies, especially those published in other languages or available as gray literature, which may lead to selection and publication bias. In addition, the validity of the included studies was not assessed, as is common in scoping review methodology (Aromataris and Munn 2020).

## Conclusions and recommendations

Our scoping review shows the paucity of currently available literature describing interventions to facilitate the use of PC and the rarity of guidelines for specific PC for this population. The majority of available studies focused on attitudes and preferences toward euthanasia, assisted suicide, and withholding or withdrawal of LSTs. The most common reasons for refusing forms of euthanasia and assisted suicide and other EOL decisions aimed at reducing patient suffering and allowing a dignified death were related to religion and religious arguments, although Islamic ethics allow some of these decisions, such as withholding or withdrawing LSTs, under certain medical conditions. In the context of PC, a focus on raising awareness of this issue among patients and health-care providers may help to improve decision-making aimed at increasing the use of PC among Muslim patients. Despite the absence of some barriers and the availability of some facilitators associated with the use of PC in non-Muslim-majority countries, there is a lack of evidence examining the participation of and benefits received by this population in this region of the world. There is a clear need for further research in this area, which should consider the facilitators associated with the use of PC and their effectiveness and practicability. We cannot draw implications for practice because the scoping review methodology does not include an assessment of the quality of the included literature (Aromataris and Munn 2020).

## Supporting information

AL Shhadat et al. supplementary material AL Shhadat et al. supplementary material

## Supplementary material

The supplementary material for this article can be found at https://doi.org/10.1017/S1478951525000148.

## References

[ref1] Abbas HA, Al Ahmadi AA, Alharby OHG, Aman RAH, Mohamed EFA, Tawlah NAK, Abbas HA, Al Ahmadi AA, Alharby OHG, Aman RAH, Mohamed EFA, and Tawlah NAK (2021) Knowledge and attitudes toward do-not-resuscitate decisions among medical students in Jeddah, Kingdom of Saudi Arabia Medical Science 25 (114), 1984–1991. https://www.discoveryjournals.org/medicalscience/current_issue/v25/n114/A24.pdf

[ref2] Abudari G, Hazeim H and Ginete G (2016) Caring for terminally ill Muslim patients: Lived experiences of non-Muslim nurses. *Palliative and Supportive Care* 14(6), 599–611. doi:10.1017/S1478951516000249 27095019

[ref3] AbuYahya O, Abuhammad S, Hamoudi B, Reuben R, Yaqub M, AbuYahya O, Abuhammad S, Hamoudi B, Reuben R and Yaqub M (2021) The do not resuscitate order (DNR) from the perspective of oncology nurses: A study in Saudi Arabia. *International Journal of Clinical Practice* 75(8), e14331. doi:10.1111/ijcp.14331 33960067

[ref4] Aghababaei N and Aghababaei N (2012) The euthanasia-religion nexus: Exploring religious orientation and euthanasia attitude measures in a Muslim context. *Omega – Journal of Death and Dying* 66(4), 333–341. doi:10.2190/OM.66.4.d 23785984

[ref5] Ahaddour C, van den B, En S and Broeckaert B (2017) Between quality of life and hope. Attitudes and beliefs of Muslim women toward withholding and withdrawing life-sustaining treatments. *Medicine, Health Care & Philosophy* 21(3), 347–361. doi:10.1007/s11019-017-9808-8 29043540

[ref6] Ahaddour C, Van den B, En S and Broeckaert B (2018) “God is the giver and taker of life”: Muslim beliefs and attitudes regarding assisted suicide and euthanasia. *AJOB Empirical Bioethics* 9(1), 1–11. doi:10.1080/23294515.2017.1420708 29267141

[ref7] Ahmed AM, and Kheir MM (2006) Attitudes towards euthanasia among final-year Khartoum University medical students. *Eastern Mediterranean Health Journal* 12(3/4), 391–397. https://applications.emro.who.int/emhj/1203_4/12_3-4_2006_391_397.pdf 17037708

[ref8] Ahmed AM, Kheir MM, Abdel Rahman A, Ahmed NH and Abdalla ME (2001) Attitudes towards euthanasia and assisted suicide among Sudanese doctors. *Eastern Mediterranean Health Journal= La Revue de Sante de la Mediterranee Orientale= Al-majallah Al-sihhiyah Li-sharq Al-mutawassit* 7(3), 551–555. doi:10.26719/2001.7.3.551 12690779

[ref9] Ahmed RA, Sorum PC and Mullet E (2010) Young Kuwaitis’ views of the acceptability of physician-assisted suicide. *Journal of Medical Ethics* 36(11), 671–676. doi:10.1136/jme.2010.036012 20817813

[ref10] Al-Awamer A and Downar J (2014) Developing a palliative care service model for Muslim Middle Eastern countries. *Supportive Care in Cancer* 22(12), 3253–3262. doi:10.1007/s00520-014-2347-4 25030938

[ref11] AlFayyad IN, Al-Tannir MA, AlEssa WA, Heena HM and Abu-Shaheen AK (2019) Physicians and nurses’ knowledge and attitudes towards advance directives for cancer patients in Saudi Arabia. *PLoS One* 14(4), e0213938. doi:10.1371/journal.pone.0213938 PMC6461283 30978182

[ref12] Al-Jahdali HH, Bahroon S, Babgi Y, Tamim H, Al-Ghamdi SM, and Al-Sayyari AA (2009) Advance care planning preferences among dialysis patients and factors influencing their decisions. *Saudi Journal of Kidney Diseases and Transplantation* 20(2), 232–239. https://journals.lww.com/sjkd/fulltext/2009/20020/Advance_Care_Planning_Preferences_Among_Dialysis.10.asp 19237810

[ref13] Almansour IM, Ahmad MM and Alnaeem MM (2020) Characteristics, mortality rates, and treatments received in last few days of life for patients dying in intensive care units: A multicenter study. *American Journal of Hospice and Palliative Medicine®* 37(10), 761–766. doi:10.1177/1049909120902976 31994897

[ref14] Almansour I, Seymour JE, Aubeeluck A, Almansour I, Seymour JE and Aubeeluck A (2019) Staff perception of obstacles and facilitators when providing end of life care in critical care units of two teaching hospitals: A survey design. *Intensive and Critical Care Nursing* 53, 8–14. doi:10.1016/j.iccn.2019.04.003 31023516

[ref15] Almuzaini AS, Salek MS, Nicholls PJ and Alomar BA (1998) The attitude of health care professionals toward the availability of hospice services for cancer patients and their carers in Saudi Arabia. *Palliative Medicine* 12(5), 365–373, doi:10.1191/026921698667234126 9924599

[ref16] Alrimawi I, Saifan AR, Abdelkader R and Batiha AM (2017) Palestinian community perceptions of do-not-resuscitation order for terminally ill patients: A qualitative study. *Journal of Clinical Nursing* 27(13), 2719–2728. doi:10.1111/jocn.13905 28557015

[ref17] Alsaati BA, Aljishi MN, Alshamakh SS, Banjar NS, Basharaheel HA, Alamri RS, Alsaati BA, Aljishi MN, Alshamakh SS, Banjar NS, Basharaheel HA and Alamri RS (2019) The concept of do not resuscitate for students in King Abdulaziz University Hospital. *Indian Journal of Palliative Care* 25(4), 544–549. doi:10.4103/IJPC.IJPC_78_19 31673210 PMC6812427

[ref18] Al-Shahri MZ (2016) Islamic theology and the principles of palliative care. *Palliative and Supportive Care* 14(6), 635–640. doi:10.1017/S1478951516000080 27109307

[ref19] Alshamsi FE, Chaaban A, Alrukhaimi M, Bernieh B, Bakoush O, Alshamsi FE, Chaaban A, Alrukhaimi M, Bernieh B, and Bakoush O (2018) Provision of renal care for patients with end stage kidney disease in persistent vegetative state, in United Arab Emirates: A national survey of renal physicians. *Libyan Journal of Medicine* 13(1), 1490610. doi:10.1080/19932820.2018.1490610 PMC6041784 29979643

[ref20] Alwadaei S, Almoosawi B, Humaidan H, Dovey S, Alwadaei S, Almoosawi B, Humaidan H and Dovey S (2019) Waiting for a miracle or best medical practice? End-of-life medical ethical dilemmas in Bahrain. *Journal of Medical Ethics* 45(6), 367–372. doi:10.1136/medethics-2018-105297 31092629

[ref21] Aramesh K, and Shadi H (2007) Euthanasia: An Islamic Ethical Perspective *Iranian Journal of Allergy, Asthma and Immunology* 6 (5), 35–38. https://www.academia.edu/399232/Euthanasia_An_Islamic_Ethical_Perspective

[ref22] Aromataris E, and Munn Z (2020) *JBI Manual for Evidence Synthesis*. Adelaide, Australien: JBI.

[ref23] Askar AHG, Ben Nakhi M, Al-Rashidi K, Al-Musabbahie BAM, Shah NM, Askar AHG, Ben Nakhi M, Al-Rashidi K, Al-Musabbahie BAM and Shah NM (2000) Physicians’ attitudes towards euthanasia in Kuwait. *Medical Principles and Practice* 9(4), 268–281. doi:10.1159/000054254

[ref24] Askarian A, Ebrahimi S, Tabei SZ, Askarian A, Ebrahimi S, and Tabei SZ (2020) The effect of Islam-based spiritual care on the quality of life in patients with breast and blood cancer. *Pakistan Journal of Medical & Health Sciences* 14(3), 1423–1425. https://pjmhsonline.com/2020/july-sep/1423.pdf

[ref25] Baeke G, Wils J-P and Broeckaert B (2012) “It’s in God’s Hands”: The attitudes of elderly Muslim women in Antwerp, Belgium, toward active termination of life. *AJOB Primary Research* 3(2), 36–47. doi:10.1080/21507716.2011.653471

[ref26] Baharoon SA, Al-Jahdali HH, Al-Sayyari AA, Tamim H, Babgi Y, and Al-Ghamdi SM (2010) Factors associated with decision-making about end-of-life care by hemodialysis patients. *Saudi Journal of Kidney Diseases and Transplantation* 21(3), 447–453. https://journals.lww.com/sjkd/fulltext/2010/21030/factors_associated_with_decision_making_about.8.aspx 20427867

[ref27] Bahramnezhad F, Cheraghi MA and Mehrdad N (2018) Do-not-resuscitate in Iranian Muslim families: A conventional content analysis. *Holist Nurs Pract* 32(5), 240–246. doi:10.1097/HNP.0000000000000284 30113957

[ref28] Bani Melhem GA, Wallace DC, Adams JA, Ross R and Sudha S (2020) Advance care planning engagement among Muslim community-dwelling adults living in the United States. *Journal of Hospice & Palliative Nursing* 22(6), 479–488. doi:10.1097/NJH.0000000000000690 33044417

[ref29] Baykara N, Utku T, Alparslan V, Arslantas MK, Ersoy N, Baykara N, Utku T, Alparslan V, Arslantas MK and Ersoy N (2020) Factors affecting the attitudes and opinions of ICU physicians regarding end-of-life decisions for their patients and themselves: A survey study from Turkey. *PLoS One* 15(5), e0232743. doi:10.1371/journal.pone.0232743 PMC7239490 32433670

[ref30] Borhani F, Hosseini SH and Abbaszadeh A (2014) Commitment to care: A qualitative study of intensive care nurses’ perspectives of end-of-life care in an Islamic context. *International Nursing Review* 61(1), 140–147. doi:10.1111/inr.12079 24382147

[ref31] Cavlak U, Aslan UB, Gurso S, Yagci N, Yeldan I, Cavlak U, Aslan UB, Gurso S, Yagci N and Yeldan I (2007) Attitudes of physiotherapists and physiotherapy students toward euthanasia: A comparative study. *Advances In Therapy* 24(1), 135–145. doi:10.1007/BF02850001 17526470

[ref32] Chamsi-Pasha H and Albar M (2017) Ethical dilemmas at the end of life: Islamic perspective. *Journal of Religion & Health* 56(2), 400–410. doi:10.1007/s10943-016-0181-3 26797682

[ref33] Clark D, Baur N, Clelland D, Garralda E, López-Fidalgo J, Connor S and Centeno C (2020) Mapping levels of palliative care development in 198 countries: The situation in 2017. *Journal of Pain and Symptom Management* 59(4), 794–807.e794. doi:10.1016/j.jpainsymman.2019.11.009 PMC7105817 31760142

[ref34] Colak D, Oguz A, Yazilitas D, Imamoglu IG, Altinbas M, Colak D, Oguz A, Yazilitas D, Imamoglu IG and Altinbas M (2014) Morphine: Patient knowledge and attitudes in the central anatolia part of Turkey. *Asian Pacific Journal of Cancer Prevention* 15(12), 4983–4988. doi:10.7314/APJCP.2014.15.12.4983 24998575

[ref35] Daar AS and Khitamy A (2001) Bioethics for clinicians: 21. Islamic bioethics. *Canadian Medical Association Journal* 164(1), 60–63. https://www.cmaj.ca/content/cmaj/164/1/60.full.pdf 11202669 PMC80636

[ref36] Duffy SA, Jackson FC, Schim SM, Ronis DL, Fowler KE, Duffy SA, Jackson FC, Schim SM, Ronis DL and Fowler KE (2006) Racial/ethnic preferences, sex preferences, and perceived discrimination related to end-of-life care. *Journal of the American Geriatrics Society* 54(1), 150–157. doi:10.1111/j.1532-5415.2005.00526.x 16420213

[ref37] Duivenbode R, Hall S, Padela AI, Duivenbode R, Hall S and Padela AI (2019) Assessing relationships between Muslim physicians’ religiosity and end-of-life health-care attitudes and treatment recommendations: An exploratory national survey. *American Journal of Hospice & Palliative Medicine* 36(9), 780–788. doi:10.1177/1049909119833335 30813738

[ref38] El Jawiche R, Hallit S, Tarabey L, Abou-Mrad F, El Jawiche R, Hallit S, Tarabey L and Abou-Mrad F (2020) Withholding and withdrawal of life-sustaining treatments in intensive care units in Lebanon: A cross-sectional survey of intensivists and interviews of professional societies, legal and religious leaders. *BMC Medical Ethics* 21(1), 1–11. doi:10.1186/s12910-020-00525-y 32859185 PMC7456082

[ref39] Farid A, Kaleybar RH, Ghobadi L, and Mousavi SR (2017) Prediction of students’ attitudes toward euthanasia using their religious orientation, self-esteem and death anxiety *Health, Spirituality and Medical Ethics* 4(3), 2–7. https://jhsme.muq.ac.ir/article-1-145-en.pdf

[ref40] Fearon D, Kane H, Aliou ND and Sall A (2019) Perceptions of palliative care in a lower middle-income Muslim country: A qualitative study of health care professionals, bereaved families and communities. *Palliative Medicine* 33(2), 241–249. doi:10.1177/0269216318816275 30554550 PMC6350179

[ref41] Fearon DM, Hughes S, Brearley SG, Fearon DM, Hughes S and Brearley SG (2021) Women’s experiences of advanced breast cancer in a resource-limited Arab context: A Stakian multi-case study. *Psycho-Oncology* 30(10), 1720–1727. doi:10.1002/pon.5735 34021523

[ref42] Gouda A, Alrasheed N, Ali A, Allaf A, Almudaiheem N, Ali Y, Alghabban A, Alsalolami S, Gouda A, Alrasheed N, Ali A, Allaf A, Almudaiheem N, Ali Y, Alghabban A and Alsalolami S (2018) Knowledge and attitude of ER and intensive care unit physicians toward do-not-resuscitate in a tertiary care center in Saudi Arabia: A survey study. *Indian Journal of Critical Care Medicine* 22(4), 214–222. doi:10.4103/ijccm.IJCCM_523_17 29743759 PMC5930524

[ref43] Hammami MM, Al Gaai E, Hammami S, Attala S, Hammami MM, Al Gaai E, Hammami S and Attala S (2015) Exploring end of life priorities in Saudi males: Usefulness of Q-methodology. *BMC Palliative Care* 14, 1–16. doi:10.1186/s12904-015-0064-5 26611147 PMC4661936

[ref44] Hammami MM, Hammami S, Amer HA, Khodr NA, Hammami MM, Hammami S, Amer HA and Khodr NA (2016) Typology of end-of-life priorities in Saudi females: Averaging analysis and Q-methodology. *Patient Preference and Adherence* 10, 781–794. doi:10.2147/PPA.S105578 27274205 PMC4876108

[ref45] Hamouda MA, Emanuel LL and Padela AI (2021) Empathy and attending to patient religion/spirituality: Findings from a national survey of Muslim physicians. *Journal of Health Care Chaplaincy* 27(2), 84–104. doi:10.1080/08854726.2019.1618063 31179903

[ref46] Hosseinzadeh K and Rafiei H (2019) Nursing student attitudes toward euthanasia: A cross-sectional study. *Nursing Ethics* 26(2), 496–503. doi:10.1177/0969733017718393 28748741

[ref47] IIF-Academy (1986) Life-Support Equipment. https://iifa-aifi.org/en/32257.html (25 March 2024).

[ref48] Islam Question & Answer (2008) Cases in which it is permissible not to use resuscitation equipment. https://islamqa.info/en/answers/115104/cases-in-which-it-is-permissible-not-to-use-resuscitation-equipment (accessed 25 March 2024).

[ref49] Iyilikci L, Erbayraktar S, Gokmen N, Ellidokuz H, Kara HC, Gunerli A, Iyilikci L, Erbayraktar S, Gokmen N, Ellidokuz H, Kara HC and Gunerli A (2004) Practices of anaesthesiologists with regard to withholding and withdrawal of life support from the critically ill in Turkey. *ACTA Anaesthesiologica Scandinavica* 48(4), 457–462. doi:10.1046/j.1399-6576.2003.00306.x 15025608

[ref50] Jansky M, Owusu-Boakye S and Nauck F (2017) Palliative care for patients with Turkish or Arabic migration background in Lower Saxony: A survey from palliative care professionals’ perspective. *Bundesgesundheitsblatt, Gesundheitsforschung, Gesundheitsschutz* 60(1), 45–54. doi:10.1007/s00103-016-2473-x 27882391

[ref51] Khalid I, Hamad WJ, Khalid TJ, Kadri M, Qushmaq I, Khalid I, Hamad WJ, Khalid TJ, Kadri M and Qushmaq I (2013) End-of-life care in Muslim brain-dead patients: A 10-year experience. *American Journal of Hospice & Palliative Medicine* 30(5), 413–418. doi:10.1177/1049909112452625 22786839

[ref52] Khalid I, Imran M, Yamani RM, Imran M, Akhtar MA and Khalid TJ (2021) Comparison of clinical characteristics and end-of-life care between COVID-19 and non-COVID-19 Muslim patients during the 2020 pandemic. *American Journal of Hospice & Palliative Medicine* 38(9), 1159–1164. doi:10.1177/10499091211018657 34039050 PMC8160924

[ref53] Mayring P and Fenzl T (2019) Qualitative inhaltsanalyse. In Baur N and Blasius J (eds), *Handbuch Methoden der Empirischen Sozialforschung*. Wiesbaden: Springer Fachmedien Wiesbaden, 633–648.

[ref54] McCABE WR and Jackson GG (1962) Gram-negative bacteremia: I. Etiology and ecology. *Archives of Internal Medicine* 110(6), 847–855. doi:10.1001/archinte.1962.03620240029006

[ref55] Muishout G, La Croix A, Wiegers G and van Laarhoven HWM (2022a) Muslim doctors and decision making in palliative care: A discourse analysis. *Mortality* 27(3), 289–306. doi:10.1080/13576275.2020.1865291

[ref56] Muishout G, Topcu N, La Croix A, Wiegers G and van Laarhoven HWM (2022b) Turkish imams and their role in decision-making in palliative care: A directed content and narrative analysis. *Palliative Medicine* 36(6), 1006–1017. doi:10.1177/02692163221095200 35848214 PMC9174576

[ref57] Muishout G, Wiegers G, Popp-Baier U and van Laarhoven HWM (2018) Muslim physicians and palliative care: Attitudes towards the use of palliative sedation. *Supportive Care in Cancer* 26(11), 3701–3710. doi:10.1007/s00520-018-4229-7 29736869 PMC6182360

[ref58] Naseh L and Heidari M (2017) The attitudes of nursing students to euthanasia. *Indian Journal of Medical Ethics* 2(1), 20–24. doi:10.20529/IJME.2017.004 27858594

[ref59] O’Neill CS, Yaqoob M, Faraj S and O’Neill CL (2017) Nurses’ care practices at the end of life in intensive care units in Bahrain. *Nursing Ethics* 24(8), 950–961. doi:10.1177/0969733016629771 26908043

[ref60] Oosterveld-Vlug MG, Francke AL, Pasman HRW and Onwuteaka-Philipsen BD (2017) How should realism and hope be combined in physician-patient communication at the end of life? An online focus-group study among participants with and without a Muslim background. *Palliative and Supportive Care* 15(3), 359–368. doi:10.1017/S1478951516000833 27819209

[ref61] Ouanes I, Stambouli N, Dachraoui F, Ouanes-Besbes L, Toumi S, Ben Salem F, Gahbiche M, Abroug F, Ouanes I, Stambouli N, Dachraoui F, Ouanes-Besbes L, Toumi S, Ben Salem F, Gahbiche M and Abroug F (2012) Pattern of end-of-life decisions in two Tunisian intensive care units: The role of culture and intensivists’ training. *Intensive Care Medicine* 38(4), 710–717. doi:10.1007/s00134-012-2500-9 22327558

[ref62] Ouzzani M, Hammady H, Fedorowicz Z, and Elmagarmid A (2016) Rayyan – a web and mobile app for systematic reviews. https://www.rayyan.ai/ (accessed 14 September 2022) 10.1186/s13643-016-0384-4 PMC5139140 27919275

[ref63] Ozcelik H, Tekir O, Samancioglu S, Fadiloglu C and Ozkara E (2014) Nursing students’ approaches toward euthanasia. *Omega* 69(1), 93–103. doi:10.2190/OM.69.1.f 25084711

[ref64] Razban F, Iranmanesh S, Aliabadi HE, Forouzi MA, Razban F, Iranmanesh S, Aliabadi HE and Forouzi MA (2016) Critical care nurses’ attitude towards life-sustaining treatments in South East Iran. *World Journal of Emergency Medicine* 7(1), 59–64. doi:10.5847/wjem.j.1920-8642.2016.01.011 27006741 PMC4786502

[ref65] Ruppert S (2019) End of life decisions. In Ruppert S and Heindl P (eds), *Palliative Critical Care: Palliative Pflegemaßnahmen auf der Intensivstation*. Berlin, Heidelberg: Springer, 99–124.

[ref66] Saeed F, Kousar N, Aleem S, Khawaja O, Javaid A, Siddiqui MF, Holley JL, Saeed F, Kousar N, Aleem S, Khawaja O, Javaid A, Siddiqui MF and Holley JL (2015) End-of-life care beliefs among Muslim physicians. *American Journal of Hospice & Palliative Medicine* 32(4), 388–392. doi:10.1177/1049909114522687 24526765

[ref67] Tricco AC, Lillie E, Zarin W, O’Brien KK, Colquhoun H, Levac D, Moher D, Peters MDJ, Horsley T, Weeks L, Hempel S, Akl EA, Chang C, McGowan J, Stewart L, Hartling L, Aldcroft A, Wilson MG, Garritty C, Lewin S, Godfrey CM, Macdonald MT, Langlois EV, Soares-Weiser K, Moriarty J, Clifford T, Tunçalp Ö and Straus SE (2018) PRISMA extension for Scoping Reviews (PRISMA-ScR): Checklist and explanation. *Annals of Internal Medicine* 169(7), 467–473. doi:10.7326/m18-0850 30178033

[ref68] Vattanavanit V, Uppanisakorn S, Bhurayanontachai R and Khwannimit B (2017) Quality of dying in the medical intensive care unit: Comparison between Thai Buddhists and Thai Muslims. *Indian Journal of Critical Care Medicine* 21(6), 59–63. doi:10.4103/ijccm.IJCCM_88_17 PMC5492738 28701842

[ref69] VERBI-Software (2022) *MAXQDA (VERBI Software)*. Berlin: VERBI Software.

[ref70] Weng RXR, Nakdali R, Almoosawi B, Al Saeed M, Maiser S, Al Banna M, Weng RX, Nakdali R, Almoosawi B, Al Saeed M, Maiser S and Al Banna M (2021) Health care providers’ attitudes and beliefs on providing palliative care to patients in Bahrain: Findings from a qualitative study. *Journal of Pain and Symptom Management* 62(1), 98. doi:10.1016/j.jpainsymman.2020.11.006 33188863

[ref71] WHO (2020a) Palliative care. https://www.who.int/news-room/fact-sheets/detail/palliative-care (accessed 18 March 2024).

[ref72] WHO (2020b) Assessing national capacity for the prevention and control of noncommunicable diseases: Report of the 2019 global survey. 9240002316. Geneva: World Health Organization. https://iris.who.int/bitstream/handle/10665/331452/9789240002319-eng.pdf (accessed 18 March 2024).

[ref73] Wolenberg KM, Yoon JD, Rasinski KA and Curlin FA (2013) Religion and United States physicians’ opinions and self-predicted practices concerning artificial nutrition and hydration. *Journal of Religion and Health* 52(4), 1051–1065. doi:10.1007/s10943-013-9740-z 23754580

[ref74] WorldAtlas (2019) Muslim population by country. https://www.worldatlas.com/articles/countries-with-the-largest-muslim-populations.html (accessed 20 March 2024).

[ref75] Yildirim JG (2020) Knowledge, opinions and behaviors of senior nursing students in Turkey regarding euthanasia and factors in Islam affecting these. *Journal of Religion & Health* 59(1), 399–415. doi:10.1007/s10943-019-00954-z 31768823

[ref76] Zafar W, Hafeez H, Jamshed A, Shah MA, Quader A and Yusuf MA (2016) Preferences regarding disclosure of prognosis and end-of-life care: A survey of cancer patients with advanced disease in a lower-middle-income country. *Palliative Medicine* 30(7), 661–673. doi:10.1177/0269216315625810 26769733

[ref77] Zamer JA and Volker DL (2013) Religious leaders’ perspectives of ethical concerns at the end of life. *Journal of Hospice & Palliative Nursing* 15(7), 396–402. doi:10.1097/NJH.0b013e31829cffa4

