# Supplementary material for: The use of palliative care by people of Islamic faith and their preferences and decisions at the end of life: A scoping review
Source: Palliat Support Care. 2025 Mar 31;23:e84. doi: 10.1017/S1478951525000148 (PMC13166356; doi:10.1017/S1478951525000148)
Supplement: AL Shhadat et al. supplementary material [file S1478951525000148sup001.docx]

| **Table 1: Description of included studies** | | | | |
| --- | --- | --- | --- | --- |
| **Study-ID** | **Objektives** | **Methods** | **Country of study** | **Population and sample size** |
| Abbas et al. 2021 | Assessment of medical students' knowledge and attitudes towards do-not-resuscitate (DNR) decisions | Cross-sectional study/online questionnaire | Saudi Arabia | 425 medical students  Inclusion criteria: medical college students  Exclusion criteria: first year students and Fakeeh Medical College students of all years of study  Female: 72.7%  Age: (mean: 22.3) years |
| Abudari et al. 2016 | Exploring the experiences of non-Muslim nurses who caring for terminally ill Muslim patients and their families, and the contexts that influence these experiences as described by the nurses. | Qualitative descriptive through interviews  The Stevick-Colaizzi-Keen method was used for data analysis. | Saudi Arabia | 10 Non-Muslim nurses  Inclusion criteria: Non-Muslim nurses with at least two years' experience of caring for terminally ill Muslim patients, able to communicate in English.  Exclusion criteria: Nurses with experience of being a patient in the hospital, Arab non-Muslim nurses.  Age (range: 29-57) years  Nationality: 3 nurses the Philippines, 2 New Zealand, and 1 each from the United Kingdom, Canada, Ireland, India and South Africa.  Specialties: 5 palliative care/oncology nurses, 3 oncology nurses and 2 medical nurses.  Years of experience in caring for terminally ill Muslim patients: (2-19) years. |
| Abu Yahya et al. 2021 | Assessment of the attitudes of oncology nurses towards DNR orders and the impact of religion on their attitudes | Cross-sectional study /questionnaire | Saudi Arabia | 157 nurses at a comprehensive cancer center  Female: 91%  Age: (mean: 34.5±6.5) years  Married: 66.7%  Religion: Muslim 19.2%, Christian 70.5%, Hindu 8.3%, other 1.9%  Oncology nursing experience: (mean: 7.3±6) years  Education level: Diploma 28.2%, Bachelor 68.6%, Master 3.2% |
| Aghababaei 2012 | Investigation of the relationship between religion and euthanasia, and comparison of single-item and multi-item scales of attitudes towards euthanasia | Cross-sectional study / questionnaire | Iran | 300 students from the University of Tehran  Female: 66%  Age: (mean: 22.6±2.5) years |
| Ahaddour et al. 2017 | 1. Exploring the attitudes and beliefs of middle-aged and older Moroccan Muslim women living in Antwerp (Belgium) towards withholding and withdrawing curative and life-sustaining treatment 2. To determine whether there is a change in attitudes and beliefs between middle-aged and older Moroccan Muslim women 3. To examine the role of religion in the attitudes 4. To show how the findings relate to the existing Islamic literature | Qualitative research/grounded theory methodology using semi-structured interviews | Belgium | 30 Moroccan Muslim women  1st group: 15 middle-aged Moroccan Muslim women; Age: (range: 41-55) years  2nd group: 15 elderly women; Age: (range: 61-86) years |
| Ahaddour et al. 2018 | 1. To investigate the relationship between contemporary normative Muslim views on assisted suicide and voluntary euthanasia on the one hand, and the actual views and attitudes of Muslims living in Belgium on the other 2. To determine whether there is a change in views and attitudes towards active euthanasia between first and second generation Muslims | Qualitative research/grounded theory methodology using semi-structured interviews | Belgium | 30 Moroccan Muslim women  1st group: 15 middle-aged Moroccan Muslim women; Age: (range: 41-55) years  2nd group: 15 elderly women; Age: (range: 61-86) years |
| Ahmed et al. 2001 | Assessment of the attitudes of Sudanese junior and senior physicians towards euthanasia and assisted suicide | Cross-sectional study/questionnaire | Sudan | 248 Sudanese physicians  Female: 48%  Age: (mean: 38±13.5) years  Religion: Muslims: 92% with moderate adherent to Islamic teachings; Christians: 8%  Education: Graduated from Sudanese universities: 72%, the remainder graduated from different Arab and Eastern European medical school |
| Ahmed and Kheir et al. 2006 | Exploration of final year medical students' attitudes towards euthanasia and identification of factors influencing their attitudes | Cross-sectional study /questionnaire | Sudan | 152 final year medical students (141 were included in analysis)  Dropout: 7.2%: 11 expressed no opinion on euthanasia  Female: 43.3%  Age: (range: 23-27) years  Religion: Muslim 100% described themselves as very religious 57.2% and as moderately religious 42.8%  Number of terminally ill patients in the last 6 months: ≥ 3 patients 33.3%, < 3 patients 66.6% |
| Ahmed et al. 2010 | Exploring the views of people in Kuwait on the acceptability of a life-ending intervention such as physician-assisted suicide | Cross-sectional study /questionnaire | Kuwait | 330 students at the College of Social Sciences  Female: 66.6%  Age: (mean: 21±1.7) years  Religion: Muslim 100% |
| Al-Awamer and Downar 2014 | Exploring the differences between PC models in Western countries and Muslim Middle Eastern countries in order to inform a culturally acceptable model of PC that meets the needs of Muslim Middle Eastern patients and their families | Qualitative empirical study | Canada | 13 English-speaking PC experts with experience in Western and Muslim Middle Eastern countries: 1 care manager and 12 physicians (6 based in the Middle East and 6 based in the West)  Female: 38.5%  Religion: Muslim 100% |
| AlFayyad et al. 2019 | Investigation of the knowledge and attitudes of physicians and nurses towards advance directives for cancer patients | Cross-sectional study/ questionnaire | Saudi Arabia | 281 Healthcare professionals (170 nurses and 111 physicians) caring for terminally ill cancer patients  Female: physicians 19.5%, nurses 86.5%  Age: physicians (mean: 33.4±7.6) years, nurses (mean: 33.9±6.6) years  Education level: physicians (Bachelor 63.3%, Master 11.9%, PhD 24.8%), nurses (Bachelor 93.1%, Master 6.9%, PhD 0%) |
| Al-Jahdali et al. 2009 | Determination of resuscitation preferences haemodialysis patients | Cross-sectional study /questionnaire | Saudi Arabia | 100 dialysis patients  Female: 45%  Age: (mean: 51.1±15.5) years  Nationality: Saudis 67%  Married: 85%  Religiosity scores: above-average 70%  Mean duration on dialysis: (6.0±4.1) years |
| Almansour et al. 2019 | Assessment of Jordanian critical care staff perceptions of the intensity and frequency of barriers and facilitators to providing end of life care (EOLC) | Cross-sectional study /questionnaire | Jordan | 104 critical care staff (76 nurses and 28 physicians)  Female: nurses 50%, Physicians 45.7%  Age (mean): nurses 26.4±2.9 years, physicians 27.2±0.96 years  Education level: nurses (Bachelor 85.5%, Master 14.5 %), physicians (Bachelor 100% Master 0%)  Years in critical care unit (mean): nurses 3.4±2.0, physicians 1.3±0.8 |
| Almansour et al. 2020 | Examining the characteristics, mortality rates and treatments received in the last days of life of patients who died in intensive care units | Retrospective multicentre cohort study | Jordan | 3885 Patients (Data from health records)  Exclusion criteria: Pediatric patients younger than 18 years and patients admitted to an intensive care unit (ICU) for less than 4 hours  Female: 43.4%  Age: (mean: 62.6±18.4) years  Multiple comorbidities: 74%  Cancer/no cancer: 85.6%/14.4%  Department transfer from: emergency department 46.8%, wards 37.7%, other hospital 10.5%, operation room 5.0% |
| Almuzaini et al. 1998 | Assessment of cancer care and hospice/PC needs of cancer patients and their carers | Cross-sectional study /questionnaire | Saudi Arabia | 695 participants (136 cancer patients,  161 informal carers, and 398 health care professionals)  Female: 37%  Age (years): patients < 25: 2.2%, 25–34: 0.7%, 35–44: 8.1%, 45–54: 31%, ≥55: 58%, informal carers: < 25: 1.2%, 25–34: 29%, 35–44: 62%, 45–54: 13%, ≥55: 0%, health care professionals: < 25: 31%, 25–34: 45%, 35–44: 21%, 45–54: 2.5%, ≥55: 0% |
| Alrimawi et al. 2017 | Presenting the views, opinions, and positions of the Palestinian community on the concept of do-not-resuscitate for terminally ill patients | Descriptive-qualitative design using semi-structured interviews | Palestinian Community | 24 participants  Female 54.1%  Age: (mean: 40,6) years  Married: 100%  Place of residence: city 33.3%, country 33.3%, refugee camp 33.3%  Educational level: primary education 16.6%, secondary education 41.6%, diploma 8.3%, bachelor or higher 33.3% |
| Alsaati et al. 2019 | Assessment of medical students' and residents' knowledge and attitudes towards the DNR order and factors influencing their attitudes | Cross-sectional study /online survey | Saudi Arabia | 429 Medical students (preclinical and clinical years)  Female: 49.9%  Age: (range: 20-25) years |
| Alshamsi et al. 2018 | Investigate nephrologists' practice of withholding and withdrawing dialysis treatment in end-of-life renal care for patients in a vegetative state | Cross-sectional study | United Arab Emirates | 29 nephrologists cared for patients with end-stage renal disease  Years of professional practice: 5-10: 13.8%, 10-20: 31%, >20: 55.2%  Profitability of dialysis unit: non-for-profit 79.3%, for-profit 20.7% |
| Alwadaei et al. 2019 | 1. Who should make decisions about withdrawing life-sustaining treatment 2. How decisions are made and the context in which decisions are made | Qualitative research design using semi-structured interviews | Bahrain | 12 medical consultants (physicians with experience in the care of brain-dead patients)  Female: 50%  Nationality: Bahraini citizens 100%  At least 15 years' experience in clinical medicine: 100%  Medical training and experience in clinical practice outside Bahrain (mainly in western countries): 100%  Contact with terminally ill patients in their clinical practice and had to make and facilitate end-of-life decisions: 100% |
| Askar et al. 2000 | Examine the effect of physician characteristics such as nationality, qualifications, years of experience, and/or religion on their attitudes towards euthanasia | Cross-sectional study /questionnaire | Kuwait | 228 physicians  Female: 27.6%  Age: (mean: 43.21±9.0) years  Nationality: Arab 40%, Kuwaiti citizens 26%, Asian 16.7%, European, American and others 16.7%  Religion: Muslim 72%, Christian 20%, Hindu/other 7%  Years of professional experience: <13: 35.1%, 14-21: 32%, 22-43: 32.9%  Place of basic medical education: Kuwait 8.3%, Arab countries 47.4%, Asian countries 16.7%, Europe, America and others 27.6% |
| Askarian et al. 2020 | Investigating the impact of Islamic PC on breast and blood cancer patients' quality of life | Before and after intervention study | Iran | 25 nurses working in oncology departments and 123 cancer patients receiving PC  Inclusion criteria: hospitalization of at least one day (24 hours), age between 18 and 60 years, willingness to participate and knowledge of their own disease, ability to report the necessary information correctly, time since diagnosis of at least two months |
| Baeke et al. 2012 | To determine how migrant women engage with the popular Western right-to-die discourse and contemporary debates about active patient euthanasia. | Qualitatives design/Grounded theory | Belgium | 30 Muslim women (15 Moroccan and 15 non-Moroccan)  Age: (range: 55-73) years  Language spoken: Turkish: all 15 women spoke Turkish as mother tongue, the Moroccans: 8 women spoke Tarifit (a Berber language, as mother tongue) and 7 women spoke Arabic  Knowledge of Dutch: All participants had no or very limited knowledge  All were first generation migrants |
| Baharoon et al. 2010 | Identify preferences for EOLC and differences in confidence in the use of cardiopulmonary resuscitation and life-sustaining measures in the event of cardiac arrest | Cross-sectional study /questionnaire | Saudi Arabia | 100 adult patients  Inclusion criteria: adult patients on haemodialysis for at least 2 years and not on the transplant list  Female 46%  Age (mean): "Certainty" response group 53.3±14.9 years, "Uncertainty" response group 46.8±16.0 years  Married:15%  Nationality: Saudi citizens 67 %, non-Saudi 33%  Working status: not working 90% |
| Bahramnezhad et al. 2018 | Exploration of the perspective of Iranian families of Muslim cancer patients on the acceptance or rejection of the DNR order | Qualitative study using content analysis | Iran | 18 participants (6 the oldest sons of the patients, 5 the husbands of the patients, 5 the fathers of the patients)  Inclusion criteria: father, first son of the family and legal guardian of terminal patients who had been told by physicians that there was no hope for them to recover their lives  Age: (mean: 40.1±12.8) years |
| Bani Melhem et al. 2020 | 1. Exploring advance care planning engagement among Muslims living in the United States 2. Examine differences in advance care planning engagement among participants according to age, gender, health status, and experience of decision-making and EOL medical treatments | Cross-sectional study /questionnaire | USA | 148 Muslim adults living in the United States  Inclusion criteria: self-identified as Muslim, adults 18 years or older, and able to read, write, and comprehend English  Female: 41.9%  Age: (mean: 36.7±13.1) years  Born in the USA: 37.8%  Country of origin: Asia 22.3%, Africa 7.4%, Arab countries 48%, Unites States 10.0%, other 8.7%  Married: 65.5%  Health status: poor 1.4%, fair 16.9%, good 35.8%, very good 31.8%, excellent 14.2% |
| Baykara et al. 2020 | Identify ICU physicians' attitudes towards EOL decisions for their patients and themselves | Cross-sectional study /online survey | Turkey | 595 ICU physicians (with at least 95% completed answers were included in the analysis)  Female: 54.5%  Age: median (range) 39 (33–45) years  Religious affiliation: believers 85.1%, undecided 5.2%, atheists 9.6%  Primary medical specialty: anesthesiology 88.2%, internal medicine 9.9%, surgery 1.9%  Years of experience: ≤2: 31.2% , 3-5: 25.3%, 6-10: 20.1%, >10: 23.4% |
| Borhani et al. 2014 | Exploring intensive care nurses' perspectives on EOLC | Descriptive qualitative design using semi-structured interviews | Iran | 12 intensive care nurses from three ICUs in teaching hospitals, using a purposive sampling technique |
| Cavlak et al. 2007 | Investigate and compare attitudes towards euthanasia among physiotherapists and physiotherapy students, and to investigate the effects of age, gender and religious affiliation on participants' acceptance of euthanasia | Cross-sectional study and comparative study/ questionnaire | Turkey | 494 participants (311 physiotherapists and 183 physiotherapy students)  Female: physiotherapists 75.9%, physiotherapy students 57.4%  Age (mean): physiotherapists 29.82±6.28 years, students 20.85±1.15 years  Religion: physiotherapists: Muslim 96.8%, Jewish 0.3%, Christian 1.3%, Atheist 1.9%, physiotherapy students: Muslim 96.8%, Atheist 3.3% |
| Colak et al. 2014 | Exploring the attitudes of cancer patients towards the use of morphine for pain management in a Muslim-majority country and identifying the factors that influence patients' decisions to accept or refuse morphine for cancer pain | Cross-sectional study /questionnaire | Turkey | 488 cancer patients  Female: 61%  Age: (median: 54, range: 18-87) Years  Primary tumour site: breast 44.4%, colorectal 19.8%, gastric 12.9% and lung 7:5%  Stage of the disease: early stage 36.8%, locally advanced 26.6, metastatic disease 35%  High school education: only 6.7% |
| Duffy et al. 2006 | Exploring EOL preferences through 10 focus groups stratified by race/ethnicity and gender | Ten focus groups and a follow-up survey | USA | 73 participants in 10 focus groups: 5 Arab Muslim women, 4 Arab Muslim men, 9 Arab Christian women , 7 Arab Christian men, 9 Hispanic women, 9 Hispanic men, 8 black women, 8 black men, 8 white women, 8 white men  Inclusion criteria: self-identification as Arab Muslim, Arab Christian, Hispanic, black, or white; English speaking; aged 50 and older; and no obvious unstable psychiatric or cognitive illness  Age: (mean: 67, range: 50–83) years |
| Duivenbode et al. 2019 | Describing the attitudes of American Muslim physicians toward EOLC decision making and to examine the associations between physician religiosity and their attitudes | Cross-sectional study /questionnaire | USA | 255 physicians  Female: 30%  Age: (mean 52.1±15.8) years  Race/Ethnicity: South Asian 69.6%, Arab/middle Eastern 21.9%  Religious affiliation with Islam: Sunni 9.9%, Shi'ite 4.5%  Years of medical practice: (mean: 19.1±14.9)  Practiced medicine in the US for over 10 years 72% |
| El Jawiche et al. 2020 | Assessment of the views and practices of intensivists in Lebanon, as well as the views of medical, legal and religious leaders regarding the withholding of life-sustaining treatment in Lebanese intensive care units | Mixed methods research: cross-section (survey) and qualitative design/interviews | Lebanon | 83 physicians  Female: 39%  Religion: Christian 68%, Muslim 22%, Druze 1%, Atheist 8%  Specialty: Critical care medicine and anesthesia 63%, critical care medicine and pulmonary medicine 34%, critical care medicine and other 3%  Average working time in ICU: permanently: 13%, ≥50%: 36%, <50%: 51%  Interviews: the head of the Lebanese order of physicians in Beirut, the head of the Lebanese Society of Anesthesiologists, and the head of the Lebanese Society of Critical Care Medicine, and the vice-president of the Lebanese national consultative committee on ethics, a representative of the medical legal opinion in Lebanon: the lawyer of Lebanese order of physicians and representatives of the main religious confessions in the country (Christian Catholic and Orthodox, Muslim Sunni and Shia, and Druze) |
| Farid et al. 2017 | Exploring the prediction of students' attitudes towards euthanasia using their religious orientation, self-esteem and death anxiety | Cross-sectional study/questionnaire | Iran | 247 students (170 from the departments of psychology and Islamic education and 77 from the Faculty of Nursing and Midwifery)  Female: 61.1% |
| Fearon et al. 2019 | Examine the congruence of palliative care principles with the perspectives of health professionals, families and communities in rural areas of the Islamic Republic of Mauritania | Qualitative design/focus groups | Mauritania | 76 participants from across rural Mauritania (33 health care professionals, 12 recently bereaved family members and 31 community leaders, in 8 focus groups and 3 semi-structured interviews) |
| Fearon et al. 2021 | Exploring the experiences of women with advanced breast cancer, their families, and health professionals in Mauritania | Qualitative/ constructivist Stakian-multi-case study approach | Mauritania | 8 women with advanced breast cancer, 10 family members 9 health professionals  Age of women with advanced breast cancer: (range 40-70) years |
| Gouda et al. 2018 | 1. Exploring the possible barriers and obstacles to the practice of DNR that could improve the process of initiating a DNR order and managing patients who have been labelled as DNR 2. Examine the impact of Islamic religion and personal beliefs on physicians' attitudes towards DNR and participating physicians' advance directives | Cross-sectional study /questionnaire | Saudi Arabia | 112 physicians (71 physicians from emergency room department and 41 physicians from ICU department)  Female: 26.8%  Age: (mean 33.06 ±7.90) years  Married: 59.8%  Religion: Muslim 97.3%, 86.6%, consider themselves reasonably religious 86.6%, non-religious 8.9%, very religious 4.5%  Years of experience ranging from 1.0 to 23  History of specialty training: trained in ICU/emergency room 79.5%, did not have formal specialty training in ICU/emergency room 20.5% |
| Hammami et al. 2015 | Exploration of Saudi men's views on end-of-life priorities and the usefulness of Q methodology in this setting | Mixed methods: exploratory cross-sectional study using of Q-methodology | Saudi Arabia | 120 participants (employees and patients at King Faisal specialist hospital and research centre, and companions of patients attending outpatient clinics)  Inclusion criteria: Saudi adults 18 years or older, who had completed at least high school education, were able to understand the purpose and procedures of the study, and provided informed consent  Age: (mean: 32.1±9.8) years  Nationality: Saudi citizens 100%  Consider their religiosity to be the same as that of other Muslims in the Kingdom of Saudi Arabia 52% |
| Hammami et al. 2016 | Exploring Saudi women's end-of-life decisions using mean analysis and Q methodology | Mixed methods: Exploratory cross-sectional study using of Q-methodology | Saudi Arabia | 68 Saudi women  Age: (mean: 30.3±8.2) years  Religiosity: much more 4%, somewhat more 31%, about the same 51%, somewhat less 9%, much less 4%  General health status: excellent 35%, very good 43%, good 16%, fair 6%, poor 0%  Life quality: excellent 28%, very good 51%, good 10%, fair 10%  High school education or more 100% |
| Hamouda et al. 2021 | 1. Describe the perspectives and practices of American Muslim physicians regarding discussions of religion and spirituality 2. How physician characteristics correlate with these perspectives | Cross-sectional study/questionnaire | USA | 255 American Muslim physicians  Female: 29.1%  Age: (mean: 52±15.7) years  Ethnicity: South Asian 70%, Arab 30%  Sect in Islam: Sunni 95.3%, Shiite 4.5%  Religiosity: their religion was the most or very important part of their lives 89%  Duration in USA: immigrated as adult 65%, immigrated as child 15.9%, born in USA 19.2%  Years of medical practice: (mean: 23.9±15.4)  Primary work setting: Clinic/office 51.6%, hospitals 48.3% |
| Hosseinzadeh and Rafiei 2019 | Exploring nursing students' attitudes towards euthanasia | Cross-sectional study/ questionnaire | Iran | 382 nursing students  Female: 61.5%  Age: (mean: 62.6±14.1) years  Religion: Muslims 100%  Clinical experience in hospitals during their college career: yes 51.8% |
| Iyilikci et al. 2004 | Exploring Turkish anaesthetists’ practices in withholding and withdrawing life support from critically ill patients | Cross-sectional study /questionnaire | Turkey | 369 anaesthesiologists  Females: 44.4%  Age: (mean 40±7.6) years  Religion: 335 Muslim, 2 Jewish, 20 atheist, 12 other  Graduation from: university hospital 78.3%, state training hospital 12.7%, social insurance training hospital 5.4%, other 35%  Years of professional experience: ≤5: 37.7%, 6-10: 27.1%, ≥11: 35.2% |
| Jansky et al. 2017 | 1. Investigation of institutional requirements for the care of people with a Turkish or Arabic background in specialized PC in Lower Saxony 2. How often is this group of patients cared for? 3. Are there specific barriers to access to specialized PC for this patient group from the providers' perspective? 4. What problem areas do PC professionals identify in the care of this patient group, and what are the responses and solutions? | Mixed methods design (survey and qualitative content analysis) | Germany | 55 health professionals working in specialist PC  Female: nurses 94.1%, social workers 100%, physicians 16.7%%  Profession: physicians 13.3%, nurses 58.3%, social workers 10.0%, other professions 16.8%, no answer 1.7% |
| khalid et al. 2013 | Assessment of EOL practices among Muslim brain-dead patients, particularly with regard to the withholding and withdrawal of therapies | Quantitative/retrospective study | Saudi Arabia | 42 brain-dead patients  Female 45.2%  Age: (mean: 46.3+19.9) years  Chronic disease in patients: none 61.9%, neoplasm 14.2%, immunosuppression, renal failure 7.1%, on dialysis 7.1%, cirrhosis 4.7%, acquired immune deficiency syndrome 4.7% |
| Khalid et al. 2021 | Investigate the clinical characteristics, EOLC and resuscitation status of Muslim patients who died due to COVID-19 and compare them with Muslim patients who died due to other diseases in 2020 | Retrospective analysis | Saudi Arabia | 96 patients (32 in the COVID-19 group and 64 in the non-COVID group)  Female: COVID-19 group 28%, non-COVID group 48%  Age: (mean: COVID-19 group: 70 ±12 years, non-COVID group: 61±16 years)  Patients with terminal disease: COVID-19 group 31%, non-COVID group 66% |
| Muishout et al. 2018 | Exploring the professional experiences of Muslim doctors using palliative sedation in relation to religious and professional norms | Qualitative design/interpretative phenomenological study using semi-structured interviews | The Netherlands | 10 Muslim physicians with professional experience in palliative sedation in general PC  Female: 20%  Religion: Muslim Sunni background and practicing 100%  Specialty: 1 trainee general practitioner, 3 general practitioner, 1 trainee neurologist, 2 trainee internist, 1 geriatrician, 1 anaesthetist, 1 acute medicine specialist  Place of education and training: fully educated and trained in the Netherlands 90% |
| Muishout & Topcu et al. 2022 | Understanding how Imams frame their role in PC decision-making / find out what roles they see for themselves and how they construct these role | Qualitative research design through direct content analysis/interview | The Netherlands | 10 Ten Turkish imams working in the Netherlands with experience in PC counselling  All men  Age: (mean 43.9 range 28-55) years  Country of birth: 9 from Turkey, 1 from the Netherlands  Level of education: 2 Master, 6 Bachelor, 1 post-secondary vocation education, 1 high school  Mean years of residence in the Netherlands: 14.8 (range 3-27) years  Years of work experience: (mean: 15.6, range 7-26) |
| Muishout & La Croix et al. 2022 | How does the language used by Muslim physicians educated in the West and working in the West shape their attitudes and practices towards palliative decision making | Qualitative/discourse analysis | The Netherlands | 10 physicians  Female 2  Ethnic background: 7 Moroccan, 2 Turkish, 1 Afghan  Religion: Muslim Sunni background and described themselves as religiously observant 100%  Specialty: 1 general practitioner in training, 3 general practitioners, 1 neurologist, 2 trainee internists, 1 geriatrician, 1 anaesthetist, 1 specialist in acute medicine, none of them were specialists in PC  Professional experience in the use of palliative sedation in a general medical setting 100% |
| Naseh and Heidari 2017 | Investigating nursing students' attitudes towards euthanasia | Cross-sectional study /questionnaire | Iran | 123 Muslim nursing students  Dropout: incomplete questionnaires 2.4%  Female: 65.8%  Age: (mean 23.1±1.6) years  Married: 31.7 %  Level of religious belief: high level 49.2%, medium level 50.8%  Economic status: low 8.3%, middle 65.8%, high25.8% |
| O'Neill et al. 2017 | Describe and identify EOLC practices that nurses contribute to | Grounded theory | Bahrain | 10 ICU nurses from two major hospitals  Inclusion criteria: Bachelor's or associate degree holders with at least 3 years of experience working in an ICU setting  Theoretical sampling  Nationality: 6 Bahraini , 4 Indian  Qualification: 4 associate degree, 6 Bachelor  Years of experience in Bahrain: range: 3.5-11 |
| Oosterveld-Vlug et al. 2017 | Investigating how people believe realistic and hopeful information should be effectively combined in physician–patient communication at the EOL | Qualitative focus group study | The Netherlands | 54 participants in three groups  1. 24 participants (patients, older people and family members without a Muslim background) in two online focus groups; group A: 13 participants; female 7; age (mean: 66.1) years and group B: 11 participants; female: 4; age (mean: 82.8) years  2. 21 Non-Muslim healthcare professionals in two online focus groups: group A: 10 participants ; female 6; age (mean:52.0) years and group B: 11 participants; female 8; age (mean: 51.9) years  3. 9 patients and carers with a Muslim background in one online focus group, female 2, age (mean: 51.7) years |
| Ouanes et al. 2012 | Report the frequency and types of EOL decisions for dying patients in two ICUs, compare medical and surgical EOL practices, and identify factors associated with EOL decisions. | Quantitative retrospective study design | Tunisia | 326 consecutive intensive care patients who died in the medical and surgical ICU over a period of 2 years  Inclusion criteria: nursing student from all years of study, willingness to participate in the study  Female 38.3%  Age (median: 64) years  McCabe: no fatal disease 73%, fatal disease (5 years) 20.3%, fatal disease (1 year) 6.7% |
| Ozcelik et al. 2014 | Exploring the attitudes of undergraduate nursing students towards euthanasia | Cross-sectional study /questionnaire | Turkey | 383 nursing students  Female: 96.9%  Age (mean: 21.3±1.5) years  Married: 3.1% |
| Razban et al. 2016 | Assessing critical care nurses' attitudes to life-sustaining treatments | Cross-sectional study /questionnaire | Iran | 884 ICU nurses  Female: 92.9%  Age: (28-35) years 58.3%  Married: 73.8%  Level of education: Bachelor 95.2%, Master 4.8%  Years of experience in ICU: <2: 27.4%, (2-5): 36.9%, >5: 35.7% |
| Saeed et al. 2015 | Exploring beliefs about aspects of EOLC among Muslim physicians in the US and other countries, and assessing the impact of different factors on DNR attitudes | Cross-sectional study /online survey | Multinational (online) | 461 physicians  Female 22.99%  Age (years): <25: 4.2%, 25-29: 16.2%, 30-39: 40.5%, 40-49: (13.6%), >50: 25.2%  Country of origin: South Asia 67.0 %, Middle East 4.9%, United States 16.9%, others 1.7%  Religion and sect: Sunni: 85.2%, Shia: 3.0%, Ahmadi: 0.6%  Specialty: medical 64.86%, surgical 13.2%, critical care 1.7%, emergency room 2.8%, radiology 1.7%  Years of experience: <5: 30.5%, 5-9: 21.4%, 10-19: 15.9%, >20: 32.1% |
| Vattanavanit et al. 2017 | Comparing the quality of dying and palliative care in the two religions, Buddhism and Islam, from the perspectives of patients, families, nurses, and physicians | Mixed methods: quantitative research design/prospective survey study | Thailand | 1. Relatives of 112 critically ill patients with chronic life‑threatening conditions and acute respiratory failure (91 Buddhists and 21 Muslims) who died after admission to a medical ICU between 2015 and 2016  Family characteristics: female: Buddhist 75.8%, Muslim 57.1%  Age: (mean: Buddhist 44.6±10.3, Muslim 42.7±10.8) years  Lived with patient: Buddhist 87.9%, Muslim 90.5%  2. The nursing staff and physicians who treated them (no further details) |
| Weng et al. 2020 | 1. Assessing the attitudes and beliefs of health care providers towards palliative care and the provision of palliative care services to the Bahraini community 2. Identify the barriers that are currently preventing the development of the speciality in the region, and future steps to address them | Qualitative study/semi-structured interviews | Bahrain | 16 Bahraini health professionals (9 physicians and 7 nurses)  Female: physicians 5, nurses all 7  Speciality: physicians (2 consultants, 6 residents and 1 senior resident), nurses (1 nurse manager, 3 head nurses and 3 nurses)  Years of experience: <10: physicians 66.7%, nurses 28.6%, ≥10: physicians 33.3%, nurses 71.4% |
| Wolenberg et al. 2013 | Investigate the relationship between physicians' religious characteristics and their approaches to artificial nutrition and hydration | Cross-sectional study /questionnaire | USA | 1156 practising US physicians  Female 35%  Religion: Muslim 10%, Jewish 10%, Hindu 7%,Roman catholic 23%, Protestant evangelical 8%, Protestant, non-evangelical 23%, other 8%, non 12%  Immigration history: born in the USA 63%, immigrated in the USA 37%  Medical school training: USA medical graduate 66%, foreign medical graduate 34%  Specialty: internal medicine 27%, family medicine 25%, cardiovascular 6%, nephrology 3%, haematology/oncology 11%, pulmonary/critical care 17%, geriatrics/hospice palliative care 12% |
| Yildirim 2020 | Examine the knowledge, opinions and attitudes of senior nursing students towards euthanasia and the factors that influence these attitudes | Descriptive correlation study /questionnaire | Turkey | 300 fourth year senior nursing students  Female: 81%  Age (years): 21-24: 97.3%, >25: 2.7%  Married: 1.3 % |
| Zafar et al. 2016 | Exploring adult cancer patients' preferences for disclosure, prognosis and EOLC | Mixed method: (cross-sectional/qualitative method (in-depth interviews)) | Pakistan | 445 cancer patients  All patients interviewed once n=420: female 58.1%, age (mean: 47.8 ±13.9) years All patients interviewed twice n=100: female 63%, age (mean: 44.2 ±11.6) years |
| Zamer and Volker 2013 | Describing the experiences of religious leaders who have supported people facing the EOL | Qualitative descriptive approach, (described by Sandelowski) | USA | 4 religious leaders from different religions (Catholicism, Judaism, Islam, and Hinduism)  Inclusion criteria: being a religious leader, defined as an ordained person of a religion and having experience in counselling people approaching the EOL  All male  Age: (range: 30-65) years  Years of experience: (range: 8-36) |


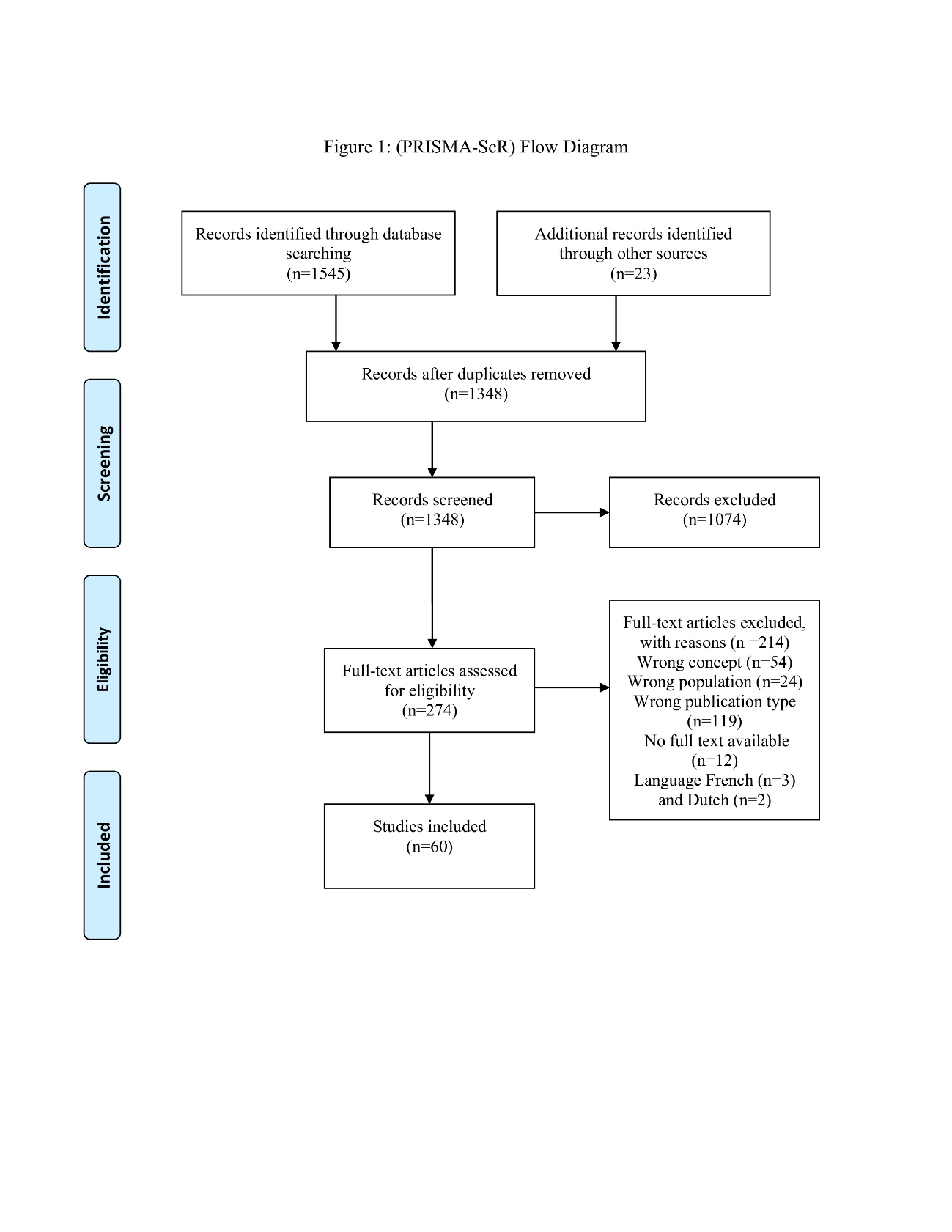


| **Table 2: Attitudes and practices** **towards EOL decisions, frequency and source study** | | | |
| --- | --- | --- | --- |
|  | **Acceptance (majority to absolute majority)** | **Refusal (majority to absolute majority)** | **Varied (no majority)** |
| **Withdrawal of one or more life-sustaining treatments or medications** | N=11  (Ahaddour et al. 2017; Alsaati et al. 2019; Baykara et al. 2020; Duivenbode et al. 2019; El Jawiche et al. 2020; Hamouda et al. 2021; Khalid et al. 2013; Oosterveld-Vlug et al. 2017; Razban et al. 2016; Yildirim 2020; Zafar et al. 2016)  Clarification:  Baykara et al 2020 had majority acceptance for withdrawal/withholding of one or more treatments, but not for non-invasive mechanical ventilation and enteral nutrition. | N=9  (Alshamsi et al. 2018; Askar et al. 2000; Borhani et al. 2014; Cavlak et al. 2007; Hosseinzadeh and Rafiei 2019; Iyilikci et al. 2004; Khalid et al. 2021; MuishoutTopcu et al. 2022; Ouanes et al. 2012)  Clarification:  By Cavlak et al. 2007 Majority refusal in both study groups (physiotherapists and physiotherapy students), but majority acceptance in the physiotherapist group | N=2  (Hammami et al. 2015; Ozcelik et al. 2014) |
| **Withholding of one or more life-sustaining treatments or medications** | N=9  (Ahaddour et al. 2017; Baykara et al. 2020; Duivenbode et al. 2019; El Jawiche et al. 2020; Gouda et al. 2018; Khalid et al. 2013; Oosterveld-Vlug et al. 2017; Yildirim 2020; Zafar et al. 2016) | N=11  (Almansour et al. 2020; Alrimawi et al. 2017; Alshamsi et al. 2018; Askar et al. 2000; Bahramnezhad et al. 2018; Cavlak et al. 2007; Hosseinzadeh and Rafiei 2019; Iyilikci et al. 2004; Khalid et al. 2021; MuishoutTopcu et al. 2022; Ouanes et al. 2012) | N=6  (Abbas et al. 2021; Al-Jahdali et al. 2009; Hammami et al. 2015; Hammami et al. 2016; O'Neill et al. 2017; Ozcelik et al. 2014) |
| **Withholding artificial nutrition or/and hydration** |  | N=5  (Almansour et al. 2020; Baykara et al. 2020; Khalid et al. 2013; Khalid et al. 2021; Wolenberg et al. 2013) |  |
| **Euthanasia (active euthanasia)** | - | N=8  (Ahaddour et al. 2018; Ahmed et al. 2001; Askar et al. 2000; Baeke et al. 2012; Cavlak et al. 2007; Hosseinzadeh and Rafiei 2019; Naseh and Heidari 2017; Yildirim 2020) | - |
| **Therapy at EOL** | N=2  (Askar et al. 2000; El Jawiche et al. 2020) | N=1  (Iyilikci et al. 2004) | - |
| **Assisted suicide** | - | N=7  (Ahaddour et al. 2018; Ahmed and Kheir 2006; Ahmed et al. 2001; Ahmed et al. 2010; Askar et al. 2000; Baeke et al. 2012; Duffy et al. 2006) | - |
| **Voluntary stopping eating and drinking** | - | - | - |
| **Palliative sedation** | N=2 * Population: physicians with experience with Terminal or palliative sedation  (El Jawiche et al. 2020; Muishout et al. 2018) | - | - |
| **Advanced care planning and advanced directives** | N=1 (AlFayyad et al. 2019) Positive attitudes toward advanced directives for cancer patients | N=1 *(no willingness to engage in ACP activities)  (Bani Melhem et al. 2020) | - |
| **Certainty about end-of-life care** | N=1 *majority with certainty regarding their preferences regarding the application of cardiopulmonary resuscitation and life sustaining measures in case of cardiac arrest  (Baharoon et al. 2010) | - | - |
| **Attitudes towards use of morphine** | - | - | N=1 *(nearly half of the patients)  (Colak et al. 2014) |
| **Hospice/palliative care** | N=3  (Almuzaini et al. 1998; Duivenbode et al. 2019; Zafar et al. 2016) | - | - |

| **Table 3: Factors and reasons for positive attitudes (acceptance) towards euthanasia, assisted suicide, or withholding/withdrawal of life-sustaining treatments, frequency and source study** |
| --- |
| Factors related to physicians and health professionals (including students of health professions) (n=16)   - Professional ethics or Professional self-concept (n=6) (Alwadaei et al. 2019; Askar et al. 2000; El Jawiche et al. 2020; MuishoutLa Croix et al. 2022; Muishout et al. 2018; Oosterveld-Vlug et al. 2017) - Gender (female) (n=3) (Ahmed and Kheir 2006; Iyilikci et al. 2004; Razban et al. 2016) - Having a clinical experience (n=2) (Cavlak et al. 2007; Hosseinzadeh and Rafiei 2019) - Euthanasia for self (n=2) (Ozcelik et al. 2014; Yildirim 2020) - Year of study (more acceptance in 3rd and 4th year) (n=1) (Hosseinzadeh and Rafiei 2019) - Atheistic viewpoint (n=1) (Baykara et al. 2020) - High level of empathy (n=1) (Hamouda et al. 2021) - Graduates of foreign schools (n=1) (Ahmed et al. 2001) - Having a Master's degree (n=1) (Razban et al. 2016) - Age (30–39 yrs.) (n=1) (Baykara et al. 2020) - Supportive nurse behaviour towards euthanasia requests (n=1) (Yildirim 2020) - Practicing of faith within the scope of science (n=1) (Alwadaei et al. 2019) |
| Principles of good death and dying (n=15):   - Autonomy and self-determination: The right to die (time and manner of death), belief in the right to euthanasia, the patient's wish and request (n=9) (Ahaddour et al. 2018; Ahmed and Kheir 2006; Ahmed et al. 2010; Baeke et al. 2012; Baykara et al. 2020; Duivenbode et al. 2019; Hosseinzadeh and Rafiei 2019; Naseh and Heidari 2017; Yildirim 2020) - Quality of life at the EOL (n=8) (Ahaddour et al. 2017; Alwadaei et al. 2019; Baeke et al. 2012; Duivenbode et al. 2019; Gouda et al. 2018; MuishoutLa Croix et al. 2022; Oosterveld-Vlug et al. 2017; Yildirim 2020) - Helping patients to die with dignity (n=2) (Ahaddour et al. 2017; Ahmed and Kheir 2006) - Ending the patient's suffering (n=2) (Ahmed and Kheir 2006; Yildirim 2020) - Reducing helplessness and hopelessness (n=1) (Yildirim 2020) - LSTs can be humiliating to the patient (n=1) (Razban et al. 2016) |
| Patient-related reasons: (n=14)   - Intolerable suffering (unbearable pain) (n=4) (Ahaddour et al. 2018; Baeke et al. 2012; Hosseinzadeh and Rafiei 2019; Muishout et al. 2018) - Imminent threat of death or imminent death (n=2) (Al-Jahdali et al. 2009; Khalid et al. 2021) - Type and severity of suffering (n=1) (Ahmed et al. 2010) - Multiple failed resuscitations (n=1) (Alrimawi et al. 2017) - Life-sustaining machines are often painful for the patient (n=1) (Razban et al. 2016) - Irreversible, fatal condition (n=1) (Baeke et al. 2012; Ozcelik et al. 2014) - Metastatic cancer not responding to treatment (n=1) (Baykara et al. 2020) - Irreversible coma (n=1) (Baeke et al. 2012) - Elderly patient over 90 years (n=1) (O'Neill et al. 2017) - Related effects (old, wanting, suffering, dependent, incurable) (n=1) (Ahmed et al. 2010) - Extreme pain and total dependency (n=1) (Ahmed et al. 2010) - In case of total dependence (n=1) (Ahmed et al. 2010) - Confirmation of brain death (n=1) (Khalid et al. 2013) - McCabe score more than 1 (n=1) (Ouanes et al. 2012) - Only if bedridden, seriously ill, unable to take medication orally (n=1) (Baeke et al. 2012) - Absence of a life prognosis for the patient (n=1) (Alrimawi et al. 2017) - Poverty (n=1) (Alrimawi et al. 2017) |
| Family related factors: (n=8)   - Burdens on the family (n=3) (Ahaddour et al. 2017; Baeke et al. 2012; Naseh and Heidari 2017) - Consent of the family (n=2) (Ahmed et al. 2001; Iyilikci et al. 2004) - Knowledge of healthcare by family members (n=1) (O'Neill et al. 2017) - Application of euthanasia by relatives to unconscious patients (n=1) (Yildirim 2020) - A kind of dedication (Give others another chance to live) (Bahramnezhad et al. 2018) |
| Medical justifications: (n=7)   - Medical futility (no benefits from these treatments) (n=3) (Ahaddour et al. 2017; Al-Jahdali et al. 2009; Alwadaei et al. 2019) - Support or agreement from two or more physicians (n=2) (Ahmed and Kheir 2006; MuishoutTopcu et al. 2022) - Approval of medical committee (n=1) (Ahmed and Kheir 2006) - Availability of alternatives to curative care (e.g. hospice care) (n=1) (Ahmed et al. 2001) - High annual proportion of terminally ill patients in ICU (n=1) (Baykara et al. 2020) - Implementation by a committee of health professionals (n=1) (Ozcelik et al. 2014) - Unavailability of ICU beds (n=1) (Baykara et al. 2020) - The need of organ donation and transplantation (n=1) (Alwadaei et al. 2019) |
| Religion and religious arguments (n=4)   - Islamic perspectives on a good death and the dying process (n=2) (Muishout et al. 2018; Saeed et al. 2015) - Theological arguments (n=2) (Ahaddour et al. 2017; Borhani et al. 2014) |
| Others: (n=3)   - Legalization of Euthanasia (n=1) (Yildirim 2020) - Personality perspectives on a good death (better to take her home to see everyone and have a peaceful passing) (n=1) (O'Neill et al. 2017) - Age (of healthy women, middle-aged) (n=1) (Ahaddour et al. 2017) |

| **Table 4: Factors and reasons for negative attitudes (refusal) towards euthanasia, assisted suicide or withholding/withdrawal of life-sustaining treatments, frequency and source study** |
| --- |
| Religion, religiosity and theological beliefs and arguments (n=25)   - Theological arguments (n=16) (Ahaddour et al. 2017; Ahaddour et al. 2018; Alrimawi et al. 2017; Baeke et al. 2012; Bahramnezhad et al. 2018; Borhani et al. 2014; Fearon et al. 2019; Fearon et al. 2021; Gouda et al. 2018; Hosseinzadeh and Rafiei 2019; MuishoutTopcu et al. 2022; Muishout et al. 2018; Oosterveld-Vlug et al. 2017; Ozcelik et al. 2014; Weng et al. 2021; Yildirim 2020) - Religiosity or high religious orientation (n=9) (Aghababaei 2012; Ahmed and Kheir 2006; Ahmed et al. 2001; Alwadaei et al. 2019; Cavlak et al. 2007; Duffy et al. 2006; Duivenbode et al. 2019; Farid et al. 2017; Naseh and Heidari 2017) - Islamic perspectives on a good death and the dying process (n=1) (Muishout et al. 2018) |
| Ethical and moral considerations (n=12)   - Ethical equivalence with suicide and murder (n=4) (Ahaddour et al. 2018; Baeke et al. 2012; Oosterveld-Vlug et al. 2017; Ozcelik et al. 2014) - Euthanasia as unethical (n=3) (Ahmed and Kheir 2006; Alrimawi et al. 2017; Ozcelik et al. 2014) - Professional ethics of physicians (valuing life beliefs) (n=2) (Muishout et al. 2018; Naseh and Heidari 2017) - Prioritizing life extension (n=2) (Naseh and Heidari 2017; Yildirim 2020) - Personal conscience and conscience issues (n=2) (Cavlak et al. 2007; Yildirim 2020) - Loss of confidence in the medical profession (n=1) (Hosseinzadeh and Rafiei 2019) - Inconsistency with the role of medicine (n=1) (Ahmed et al. 2001) - Moral obligation (n=1) (Oosterveld-Vlug et al. 2017) |
| Medical and practical considerations (n=10)   - Faith in medicine to cure disease (n=3) (Ahaddour et al. 2017; Fearon et al. 2019; Oosterveld-Vlug et al. 2017) - Fear of biasing future research away from better care (n=2) (Ahmed and Kheir 2006; Ahmed et al. 2001) - Dependence on future medical developments (n=2) (Cavlak et al. 2007; Yildirim 2020) - Possibility of improving patient's health (n=1) (Alrimawi et al. 2017) - Presence of subtle pressure on patients, fear of dependency or humiliation (n=1) (Ahmed et al. 2001) - Possibility of misdiagnosis of brain death due to no good evidence (n=1) (Alwadaei et al. 2019) - Damage to patient-nurse relationships (n=1) (Hosseinzadeh and Rafiei 2019) - In the case of a tolerable pain (n=1) (Ahaddour et al. 2017) |
| Family related factors (n=8)   - Family refusal (n=7) (Alwadaei et al. 2019; Bahramnezhad et al. 2018; Iyilikci et al. 2004; MuishoutLa Croix et al. 2022; Muishout et al. 2018; O'Neill et al. 2017; Oosterveld-Vlug et al. 2017) - Lack of medical awareness in the family (n=2) (Alwadaei et al. 2019; Muishout et al. 2018) - Family obligations to continue treatment (n=1) (Alwadaei et al. 2019) - Doubts about health professional's knowledge of treatment (n=1) (Fearon et al. 2019) - Forcing of feelings (n=1) (Bahramnezhad et al. 2018) |
| Physician-related factors (n=6)   - Unfamiliarity with PC and PC treatment possibilities (n=2) (Muishout et al. 2018; Oosterveld-Vlug et al. 2017) - Physician support for false hope (n=1) (Oosterveld-Vlug et al. 2017) - Factors related to the size of the medical center and the ICU (n=1) (Iyilikci et al. 2004) - Unfamiliar with the concept of euthanasia (n=1) (Ahmed and Kheir 2006) - Unclear or poor communication from physicians (n=1) (Oosterveld-Vlug et al. 2017) - Lack of courage (n=1) (Cavlak et al. 2007) - Preference for coma over euthanasia (n=1) (Hosseinzadeh and Rafiei 2019) |
| Patient related factors (n=5)   - Refusal of patient or specific request (n=4) (Ahaddour et al. 2017; Bahramnezhad et al. 2018; Hammami et al. 2015; Oosterveld-Vlug et al. 2017) - Having an independent functional status prior to ICU (n=1) (Ouanes et al. 2012) - A difficult farewell to family and life (n=1) (Ahaddour et al. 2017) |
| Social and cultural factors (n=5)   - Clergy opposition (n=2) (Bahramnezhad et al. 2018; El Jawiche et al. 2020) - Effect of community spiritual healers (n=1) (Al-Awamer and Downar 2014) - Social obligation to fight disease and not to give up (n=1) (Oosterveld-Vlug et al. 2017) - Local culture (n=1) (Alwadaei et al. 2019) |
| Legal concerns and regulatory issues (n=4)   - Fear of potential misuse Use by incompetent people (n=2) (Ahmed and Kheir 2006; Ahmed et al. 2001) - Legal obligations (n=1) (Yildirim 2020) - Lack of laws and policies (n=1) (Alwadaei et al. 2019) - Loss of mental competence (n=1) (Ahmed et al. 2001) - Fear of lawsuits (n=1) (Alwadaei et al. 2019) - Fear of non-compliance (n=1) (Ahmed et al. 2001) - Euthanasia as illegal (n=1) (Yildirim 2020) |
| Others (n=6)   - Age (older) (n=2) (Ahaddour et al. 2017; Ozcelik et al. 2014) (Immigrants women and nursing students respectively) - Individual definition of a good death (doing everything possible) (n=1) (Fearon et al. 2019) - Personal extrinsic orientation/ university students (n=1) (Aghababaei 2012) - Gender (female) (n=1) (Naseh and Heidari 2017) - Age (younger nursing students) (n=1) (Naseh and Heidari 2017) - Arabic ethnicity (n=1) (Hamouda et al. 2021) - Study year (negative correlation) (n=1) (Ozcelik et al. 2014) |

| **Table 5: Others should be involved in the decision-making process** | |
| --- | --- |
| **Other types** | **Frequency and source study** |
| An ethics committee (significant portion)  An ethics committee (Majority)  Religious authorities (portion not stated)  Other secular higher authorities (portion not stated)  Society (portion not stated)  Consensus of different Participants (majority)  A religious advisor (significant portion) | 1 (Baykara et al. 2020)  1 (El Jawiche et al. 2020)  1 (Alwadaei et al. 2019)  1 (Alwadaei et al. 2019)  1 (Alwadaei et al. 2019)  1 (Iyilikci et al. 2004)  1 (Askar et al. 2000) |

| **Table 6:**  **Facilitators associated with the use of PC, frequency and source study** |
| --- |
| Facilitators at hospital level (n=7)   - Explaining opioid therapy to families by palliative care physicians (n=1) (Abudari et al. 2016) - Collaborating within healthcare team (n=1) (Borhani et al. 2014) - Ensuring availability of fully equipped hospice care support (n=1) (Almuzaini et al. 1998) - Enhancing psychosocial support programs (n=1) (Al-Awamer and Downar 2014) - Engaging nurses/other health teams in building the PC services (n=1) (Al-Awamer and Downar 2014) - Prioritizing continuity of care for dying patients (n=1) (Al-Awamer and Downar 2014) - Building relationships within health care team (n=1) (Al-Awamer and Downar 2014) - Getting administration support (n=1) (Al-Awamer and Downar 2014) - Choosing the “right” personnel (n=1) (Al-Awamer and Downar 2014) - Promote positive attitudes towards PC among health (n=1) (Almuzaini et al. 1998) - Supporting non-licensed staff to assist in the care of dying patient (n=1) (Almansour et al. 2019) - Talking with the patient about his feelings and thoughts about (n=1) (Almansour et al. 2019) - Allowing families unlimited access to the dying patient (n=1) (Almansour et al. 2019) - Having an ethics committee member (Nurses) (n=1) (Almansour et al. 2019) - Availability of professional opinion of three specialized reliable physicians (El Jawiche et al. 2020) - Having the physicians agree about the direction care (n=1) (Almansour et al. 2019) - Allowing continuity of care for the dying patient by the same nurse (n=1) (Almansour et al. 2019) - Effective pain management in PC (n=1) (Jansky et al. 2017) |
| Organizational strategies at a regional/national level (n=4)   - Develop or have national PC health policies and guidelines (n=2) (Al-Awamer and Downar 2014; El Jawiche et al. 2020) - Enhancing psychosocial support programs within hospitals (n=1) (Oosterveld-Vlug et al. 2017) - Improve access to PC medication and services (n=1) (Al-Awamer and Downar 2014) - Integrate PC programs across a region/nation (n=1) (Al-Awamer and Downar 2014) - Establishment of a hospice, fully equipped with specialist staff (n=1) (Almuzaini et al. 1998) |
| Facilitators related to patients and their families (n=4)   - Facilitating discussions with all parties or via a family spokesperson (n=1) (Jansky et al. 2017) - Permission from family to “stop life support” (n=1) (Borhani et al. 2014) - Acceptance of the reality of terminal illness among family members (n=2) (Abudari et al. 2016; Almansour et al. 2019) - Allowing the family to help physically care for the dying patient (n=1) (Almansour et al. 2019) |
| Facilitators related to health professionals (n=3)   - Emphasize respect for humanity and dignity (n=2) (Borhani et al. 2014; Oosterveld-Vlug et al. 2017) - Having a good communication skills (n=2) (Fearon et al. 2019; Oosterveld-Vlug et al. 2017) - Professional obligation to provide care until the last moment of the patient's life (n=1) (Borhani et al. 2014) - Having emotional relationship with patients (n=1) (Borhani et al. 2014) |
| Facilitators related to communication and interaction between patients/families and health care professionals (n=3)   - Recognition of end-of-life phase by an external person (physicians) (n=1) (Fearon et al. 2019) - Existing of consent signed by family and physicians (n=1) (El Jawiche et al. 2020) - A pragmatic approach to conflict resolution strategies (n=1) (Jansky et al. 2017) - Offering dialogue to resolve conflicts (n=1) (Jansky et al. 2017) - Provision of information material in foreign languages (n=1) (Jansky et al. 2017) - Building trust (n=1) (Jansky et al. 2017) - Repeated dialogue about the patient's situation and therapy goals (n=1) (Jansky et al. 2017) - Communication using picture boards or books (n=1) (Jansky et al. 2017) - Providing access to translators (n=1) (Jansky et al. 2017) |
| Integration of religious and cultural practices in PC (n=2)   - Involving a Muslim spiritual counsellor to facilitate clarity in clarity in communication (n=1) (Oosterveld-Vlug et al. 2017) - Existence of religious guidelines by Sunni Muslims (n=1) (El Jawiche et al. 2020) |
| PC education and training and research (n=2)   - Expanding PC training programs for medical students and professionals (n=2) (Al-Awamer and Downar 2014; Jansky et al. 2017) - Educating medical staff about PC principles and practices (n=1) (Al-Awamer and Downar 2014) - Training and material for the care of people with migration background (n=1) (Jansky et al. 2017) - Supporting PC researches (n=1) (Al-Awamer and Downar 2014) |
| Enhancing patient-centred care/respecting patient preferences (n=1) (Jansky et al. 2017)   - Acceptance of differences and recognition of each patient as an individual - Assignment of a same-sex health care provider - Optimization of care (enabling people to perform religious rituals such as washing and praying) |
| Societal facilitators (n=1) (Al-Awamer and Downar 2014)   - Involving community and religious leaders in PC advocacy - Improving public awareness |

| **Table 7: Barriers associated with the use of PC, source study and frequency** |
| --- |
| Barriers related to patients and families and their behaviour (n=10)   - Lack of information and awareness about the roles and benefits of PC (n=3) (Almansour et al. 2019; Almuzaini et al. 1998; Jansky et al. 2017) - Rejection of family(Al-Awamer and Downar 2014) (n=3) (Almansour et al. 2019; Jansky et al. 2017; Muishout et al. 2018) - Intra-family conflicts on the direction of therapies (n=2) (Almansour et al. 2019; Jansky et al. 2017) - Aggressive behaviours and dealing with angry or distraught family members (n=2) (Almansour et al. 2019; Jansky et al. 2017) - Problematic symptoms or psychosocial situation (n=2) (Almansour et al. 2019; Jansky et al. 2017) - Reluctance to report pain (n=1) (Colak et al. 2014) - Unwillingness to use opioids due to ‘myths about opioids’ (n=1) (Colak et al. 2014) - Mistrust of outsiders (healthcare providers) (n=1) (Colak et al. 2014) - Difficulties discussing with patients and families (n=1) (Weng et al. 2021) - Financial status of the family (n=1) (Fearon et al. 2019) - Rejection of care in general or specific forms from care services (resistance to use PC) (n=1) (Jansky et al. 2017) - Isolation of families with migration background (n=1) (Jansky et al. 2017) - Potential existence, social network that takes care of them (n=1) (Jansky et al. 2017) - Use of alternative structures (specialised care service for migrants) (n=1) (El Jawiche et al. 2020) - Families’ unrealistically high expectations (n=1) (Borhani et al. 2014) - Systems of Families (many family members are obliged to visit (n=1) (Jansky et al. 2017) - The family is not present with the dying patient (n=1) (Almansour et al. 2019) - Continuous calls to physicians from family members (n=1) (Almansour et al. 2019) - Interrupting work routines and disrupting the physical comfort of patients because of a religious obligation (non-Muslim nurses), (n=1) (Abudari et al. 2016) |
| Laws and policies (n=8)   - Legal concerns and restrictions (n=6) (Al-Awamer and Downar 2014; Almansour et al. 2019; Borhani et al. 2014; Colak et al. 2014; El Jawiche et al. 2020; Weng et al. 2021) - Absence of laws, policies, regulatory frameworks and guidelines (n=6) (Al-Awamer and Downar 2014; Almansour et al. 2019; Alwadaei et al. 2019; Borhani et al. 2014; El Jawiche et al. 2020; Weng et al. 2021) - Challenges in implementation of PC policies (n=2) (Al-Awamer and Downar 2014; Colak et al. 2014) - No financial benefit in the governmental or private sector (n=1) (Almuzaini et al. 1998) |
| Lack of education, knowledge, and exposure (n=7)   - Lack of familiarity with the role and benefits of PC (n=2) (Al-Awamer and Downar 2014; Jansky et al. 2017) - Lack of knowledge and training in pain management (n=2) (Borhani et al. 2014; Colak et al. 2014) - Limited exposure of medical trainees to PC and EOL issues (n=1) (Al-Awamer and Downar 2014) - Limited practical experience (n=1) (Fearon et al. 2019) - Lack education and training for clinicians (n=1) (Almansour et al. 2019) - Lack of education in society (n=1) (Weng et al. 2021) |
| Structure of the health care system (n=6)   - Access problems (n=3) (Al-Awamer and Downar 2014; Fearon et al. 2019; Jansky et al. 2017) - Continuing of treatment due to financial benefits to the hospital (n=2) (Al-Awamer and Downar 2014; Almansour et al. 2019) - Limited availability of hospice care (n=2) (Al-Awamer and Downar 2014; Weng et al. 2021) - Lack of a national integrated and accessible PC service (n=1) (Al-Awamer and Downar 2014) - Lack of support programmes for PC professionals (n=1) (Al-Awamer and Downar 2014) - Hierarchy within the administration and within medical team (n=1) (Al-Awamer and Downar 2014) - Limited integration of services across care settings (n=1) (Al-Awamer and Downar 2014) - Unsafe environment (n=1) (Jansky et al. 2017) - Problems by organization and planning of care (care provider from other gender) (n=1) (Jansky et al. 2017) - Poor unit design that does not allow privacy for dying patients (n=1) (Almansour et al. 2019) - Lack of government support for PC programmes (n=1) (Al-Awamer and Downar 2014) - Lack of administrative support for PC at hospital level (n=1) (Al-Awamer and Downar 2014) - Lack of support for family (social worker/religious leader) (n=1) (Almansour et al. 2019) - Uncomfortable environment for Muslim women (n=1) (Duffy et al. 2006) - Use of alternative structures (specialized migrant care services) (n=1) (Jansky et al. 2017) - Problems with visiting hours (too restrictive or too generous) (n=1) (Almansour et al. 2019) |
| Barriers related to cultural norms and values (n=7)   - Dealing with the cultural differences (n=4) (Abudari et al. 2016; Almansour et al. 2019; El Jawiche et al. 2020; Jansky et al. 2017) - Cultural barriers related to end-of-life care (n=2) (Duffy et al. 2006; Jansky et al. 2017) - No acceptance of the loss of patient (n=1) (Weng et al. 2021) - Truth-telling blocked by family (n=1) (Al-Awamer and Downar 2014) |
| Barriers to communication and interaction between patients, relatives and health care professionals (n=6)   - Communication barriers in/during medical consultations (n=1) (Fearon et al. 2019) - Inability to communicate with others due to coma (n=1) (Borhani et al. 2014) - No space for questions (n=1) (Fearon et al. 2019) - Giving a little or incorrect information (n=1) (Fearon et al. 2019) - Lack or limited cognitive ability of the patient (n=1) (Jansky et al. 2017) - Lack of language skills when interacting with patient and family (n=1) (Jansky et al. 2017) - Different gender understanding (n=1) (Jansky et al. 2017) - Problems in making contact (n=1) (Jansky et al. 2017) - Language barriers (n=2) (Abudari et al. 2016; Jansky et al. 2017) - Avoiding conversations with family (n=1) (Almansour et al. 2019) - Unclear or poor communication from physicians (n=1) (Oosterveld-Vlug et al. 2017) |
| Barriers related to behaviour of health care professionals (n=6)   - Non-involvement of palliative care team due to lack of referral (n=1) (Abudari et al. 2016) - Ethical conflicts due to different wishes of team/patient and family (n=1) (Jansky et al. 2017) - Clinicians overly optimistic about patient survival (n=1) (Almansour et al. 2019) - Clinicians who focusing on saving the patient's life (not the quality of EOL) (n=1) (Almansour et al. 2019) - Clinicians who won’t allow the patient to die (n=1) (Almansour et al. 2019) - Clinicians know about the patient's poor prognosis before family (n=1) (Almansour et al. 2019) - Do not seek other opinions from other healthcare professionals (n=1) (Almansour et al. 2019) - Providing LST requested by family, despite patient's signed advanced directive (n=1) (Almansour et al. 2019) - Not knowing or seeking the patient's wishes (n=1) (Almansour et al. 2019) - Continuing treatment of dying patient, despite pain and discomfort (n=1) (Almansour et al. 2019) - Avoiding discussion with family (n=1) (Almansour et al. 2019) - Misperception about health/dealing with morbidity/mortality (n=1) (Colak et al. 2014) - Resistance of medical staff to PC and late referral (n=1) (Al-Awamer and Downar 2014) - Physicians not interested in working in this area (n=1) (Almuzaini et al. 1998) - Disagreement between multiple clinicians about direction of care (n=1) (Almansour et al. 2019) |
| Lack of necessary resources (n=4)   - Lack of qualified medical staff (n=3) (Al-Awamer and Downar 2014; Borhani et al. 2014; Weng et al. 2021) - Limited availability of medication (opioids) (n=2) (Al-Awamer and Downar 2014; Colak et al. 2014) - Limitations in establishing a functional multidisciplinary team (n=1) (Al-Awamer and Downar 2014) - Limited support for PC research (n=1) (Al-Awamer and Downar 2014) - Limited financial resources (n=1) (Al-Awamer and Downar 2014) - Lack of integrated patient-centred spiritual care (n=1) (Al-Awamer and Downar 2014) - Limited psychosocial support(n=1) (Al-Awamer and Downar 2014) |
| Social pressure (Fear of societal judgment and stigma) (n=2) (Fearon et al. 2019; Weng et al. 2021) |
| Religious beliefs (n=2) (Borhani et al. 2014; Weng et al. 2021) |

# **Review appendices**

**Appendix 1: Search strings**

| **Pubmed** | | |
| --- | --- | --- |
| **Search strategies** | **Number of search results** | **Time frame** |
| 1. “Muslim” [MeSh Terms] 2. “Muslims”[MeSh Terms] 3. “Islam”[MeSh Terms] 4. “Arab”[MeSh Terms] 5. “Mohammedanism”[MeSh Terms] 6. **1 OR 2 OR 3 OR 4 OR 5** 7. **“Treatmen refusal**”[MeSh Terms] 8. “right to die”[MeSH Terms] 9. “assisted suicide”[MeSH Terms] 10. “euthanasia”[MeSH Terms] 11. “analgesia”[MeSH Terms] 12. “deep sedation”[MeSH Terms] 13. “pain management”[MeSH Terms] 14. “withholding resuscitation”[MeSH Terms] 15. “withholding treatment”[MeSH Terms] 16. “advance care planning”[MeSH Terms] 17. “advance medical planning”[MeSH Terms] 18. “attitude to death”[MeSH Terms] 19. “decision making”[MeSH Terms] 20. “decision support models”[MeSH Terms] 21. “terminally ill”[MeSH Terms] 22. “life support care”[MeSH Terms] 23. “palliative care”[MeSH Terms] 24. “palliative medicine”[MeSH Terms] 25. “bereavement care”[MeSH Terms] 26. “hospice care”[MeSH Terms] 27. **7 OR 8 OR 9 OR 10 OR 11 OR 12 OR 13 OR 14 OR 15 OR 16 OR 17 OR 18 OR 19 OR 20 OR 21 OR 22 AND 23 OR 24 OR 25 OR 26** 28. **6 AND 27** | 542 | 1.8.2022-31.8.2022 |

**Appendix 2: Data extraction sheet**

| Study ID (author, publication date) |  |
| --- | --- |
| Study title |  |
| Country of origin of the study |  |
| Methodology and type of evidence (design) |  |
| Aims |  |
| Population and sample size |  |
| Study concept |  |
| Context and setting |  |
| Interventions (including details such as duration of intervention) |  |
| Comparators / comparison group if available |  |
| Measurement instruments |  |
| Results and conclusions in relation to the scoping review questions (detailed) |  |
| Notes |  |
